# Effect of IL-27, Teriflunomide and Retinoic Acid and Their Combinations on CD4^+^ T Regulatory T Cells—An In Vitro Study

**DOI:** 10.3390/molecules27238471

**Published:** 2022-12-02

**Authors:** Tomasz Maślanka

**Affiliations:** Department of Pharmacology and Toxicology, Faculty of Veterinary Medicine, University of Warmia and Mazury in Olsztyn, Oczapowskiego Street 13, 10-719 Olsztyn, Poland; tomasz.maslanka@uwm.edu.pl; Tel.: +48-89-524-6145

**Keywords:** IL-27, teriflunomide, ATRA, Treg cells, Tr1 cells

## Abstract

The principal goal of the study was to verify the concept of pharmacological induction of Foxp3^+^CD25^+^CD4^+^ T regulatory (Treg) cells which will additionally be characterized by a highly suppressive phenotype, i.e., by extensive CD25 and CD39 expression and IL-10 and TGF-β production. Stimulated and unstimulated murine lymphocytes were exposed to IL-27, teriflunomide (TER), and all trans retinoic acid (ATRA) alone and to their combinations. The study demonstrated that: (a) IL-27 alone induced CD39 expression on Treg cells and the generation of Tr1 cells; (b) TER alone induced Foxp3-expressing CD4^+^ T cells and up-regulated density of CD25 on these cells; TER also induced the ability of Treg cells to TGF-β production; (c) ATRA alone induced CD39 expression on Treg cells. The experiments revealed a strong superadditive effect between IL-27 and ATRA with respect to increasing CD39 expression on Treg cells. Moreover, IL-27 and ATRA in combination, but not alone, induced the ability of Treg cells to IL-10 production. However, the combination of IL-27, TER, and ATRA did not induce the generation of Treg cell subset with all described above features. This was due to the fact that TER abolished all listed above desired effects induced by IL-27 alone, ATRA alone, and their combination. IL-27 alone, ATRA alone, and their combination affected TER-induced effects to a lesser extent. Therefore, it can be concluded that in the aspect of pharmacological induction of Treg cells with a highly suppressive phenotype, the triple combination treatment with TER, IL-27, and ATRA does not provide any benefits over TER alone or dual combination including IL-27 and ATRA.

## 1. Introduction

Immune tolerance is an important mechanism counteracting the development of allergies and autoimmune diseases. Foxp3^+^CD25^+^CD4^+^ T regulatory (Treg) cells play a critical role in establishing and maintaining self-tolerance and immune homeostasis [1]. Research evidence indicates that deficiency of Treg cells or their defective functioning are related to the immunopathogenesis of multiple autoimmune diseases and inflammatory conditions, whereas expansion of Treg cells and/or amelioration in their functions are associated with recovery from autoimmune diseases [2,3]. Treg cells can be divided into two principal subsets: naturally occurring Tregs (nTregs) and induced (also called inducible or adaptive) Tregs (iTregs). nTregs develop in the thymus [1]. In turn, iTregs are induced de novo in the periphery from naïve Foxp3^−^CD25^−^CD4^+^ T cells, and can be generated in vitro following antigenic stimulation in the presence of TGF-β and IL-2 [4]. The transcription factor Foxp3 is not only a marker used to distinguish Treg cells (i.e., Foxp3^+^CD25^+^CD4^+^) from activated effector (aTeff) CD4^+^ T cells (i.e., Foxp3^−^CD25^+^CD4^+^), but it actually confers the regulatory activity of Tregs because it is critically involved in the development and function of these cells [5]. Loss of Foxp3 expression or function can lead to the development of autoimmune diseases [6,7], while ex vivo generated iTregs from Foxp3-negative CD4^+^ Teff cells exhibit the suppressive and phenotypic characteristics like nTreg [8,9,10]. Treg cells suppress immune responses through multiple mechanisms, which can be broadly divided into those that target T cells (suppressor cytokines, IL-2 consumption, cytolysis) and those that primarily target antigen-presenting cells (decreased costimulation or decreased antigen presentation) [11]. CD39 (ectonucleoside triphosphate diphosphohydrolase-1; ENTPD1) is a very important surface molecule associated with Treg cell function because the production of adenosine via the CD39/CD73 (ecto-5′-nucleotidase; 5′-N) pathway is recognized as a major mechanism responsible for the immunosuppressive function of Treg cells [12]. Binding of adenosine to A2A receptors on T cells leads to the accumulation of intracellular cAMP, which subsequently prevents upregulation of CD25 on Teff cells and suppresses proliferation of Teff cells and production of pro-inflammatory cytokines [12,13]. Dysregulation of CD39 expression appears to be crucially involved in various human pathologies. Reduced CD39 expression has been demonstrated in certain autoimmune diseases, while elevated expression of this molecule has been found on tumor-infiltrating Treg cells in various malignancies [14]. Another important mechanism through which Treg cells suppress T cell-mediated immune responses is the production IL-10 and TGF-β, i.e., the major anti-inflammatory and immunosuppressive cytokines. Generally, IL-10 exerts immunosuppressive effects on various cell types [15]. The key role played by IL-10 and TGF-β produced by Treg cells during the generation of immune tolerance to allergens is well established [16]. The results of several studies indicate that TGF-β produced by Treg cells is required to inhibit allergic and autoimmune inflammatory responses [17,18,19,20]. In turn, IL-10 produced by Treg cells plays an instrumental role in limiting inflammation especially at the mucosal interface [21].

IL-10 producing type 1 regulatory T (Tr1) cells constitute another subset of CD4^+^ regulatory T cells, which also plays a crucial role in promoting and maintaining immunotolerance and thereby in preventing allergic and autoimmune diseases [22]; they do not express Foxp3 but express surface markers LAG3 (CD223) and CD49b [23]. In contrast to Treg cells, Tr1 cells do not occur naturally as such, but they are induced in the periphery upon chronic antigen stimulation in the presence of IL-10 derived from tolerogenic antigen-presenting cells [24].

Taking the above into account, it is unsurprising that Treg and Tr1 cells represent an attractive and promising therapeutic approach to treatment of autoimmune and allergic disorders [25,26,27]. Therefore, the induction of generation of iTreg cells and/or reinforcement function of Treg cells may constitute a potential treatment in patients with such diseases. This is manifested in the literature, as numerous papers have been published in the recent years strongly suggesting that a specific drug or a candidate for a drug induces the generation of iTreg cells and/or upregulates the expression of some immunosuppressive molecules in Treg cells [28,29,30,31,32,33]. A preferred solution in contemporary medicine is polytherapy, that is a combination therapy where several drugs with the same direction, but different mechanisms of action are administered, which—owing to the phenomenon known as superadditivity—allows the enhancement of the therapeutic effect with low doses of the drugs. The concept of polytherapy seems to be of particular importance in the context of pharmacological induction of iTreg cells. Due to the complexity of the mechanism of action of these cells, it is highly unlikely that that a single drug could both generate iTreg—via induction of the Foxp3 expression in conventional CD4^+^ T cells—and increase the expression/production of immunosuppressive molecules associated with Treg cell function (e.g., CD25, CD39, IL-10, and TGF-β). Hence, a concept was put forth in this study of the de novo generation of iTreg cells with a highly suppressive phenotype (‘Super Tregs’) with the help of combined application of several drugs or other agents. The essence of this concept is that individual compounds: (a) will complement each other, i.e., they will upregulate different immunosuppressive molecules/attributes of Treg cells; (b) together will exert a superadditive or at least an additive effect with respect to the upregulation of particular immunosuppressive molecules and the number of iTreg cells. Therefore, the principal goal of this study was to verify the concept of the pharmacological induction of iTreg cells which will additionally be characterized by a highly suppressive phenotype, i.e., by extensive CD25 and CD39 expression and IL-10 and TGF-β production. This was verified by means of a combined treatment with IL-27, teriflunomide (TER), and all trans retinoic acid (ATRA). Moreover, the effect of these agents and their combinations on the induction of Tr1 cells was also determined.

IL-27 is a multifaceted heterodimeric cytokine with pronounced pro- and anti-inflammatory as well as immunoregulatory functions [34]. IL-27 is involved in T cell differentiation, inflammation, and infections and shows overlapping activities and plasticity with IL-6 and IL-35 [34]. Several studies demonstrated that IL-27 may be considered as a potential therapeutic agent for some autoimmune or inflammatory diseases, including, among others, colitis [35], multiple sclerosis [36], or rheumatoid arthritis [37].

TER is an immunosuppressive drug with anti-inflammatory properties that inhibits the mitochondrial enzyme dihydroorotate dehydrogenase in proliferating cells, leading to inhibition of de novo pyrimidine synthesis and consequently reduced proliferation of T and B cells [38,39]. However, the mode of action of TER does not seem to have been fully elucidated yet. TER is approved for the treatment of mild to moderate relapsing–remitting multiple sclerosis, whereas its precursor leflunomide is in clinical use for the treatment of rheumatoid arthritis and psoriatic arthritis [40,41].

ATRA, a vitamin A derivative, is a signaling molecule with significant roles in growth, differentiation, and apoptosis in different tissues [42]. The effects of ATRA are mediated by specific nuclear receptors, the retinoic acid receptor and the retinoid X receptor, which belong to steroid/thyroid hormone receptor superfamily [43]. ATRA is a promising drug that has shown potential therapeutic effects in various cancers [44].

The selection of IL-27, TER, and ATRA followed from the literature perusal as well as a preliminary study carried out before. In light of these investigations, the effects that support and fit into the essence of the research concept presented above can be put forth as follows: (a) exposure to IL-27 should lead to generation of Tr1 cells [45,46,47] and can expand IL-10-producing [48] and CD39-expressing (own unpublished observation) Treg cells; (b) exposure to TER should lead to induction of the generation of iTreg cells—as a result of the induction of Foxp3 expression in conventional CD4^+^ T cells [33,49]—and Tr1 cells [50], and expand TGF-β-producing Treg cells [51]; (c) exposure to ATRA may promote conversion of conventional CD4^+^ T cells into iTreg cells [52,53,54] and increase CD39 expression on Treg cells (own unpublished observation).

However, it is known that while combined application of drugs may cause superadditivity or additivity, it may also lead to the emergence of antagonism, that is, the abolition of the effect of one drug by another one. Therefore, the intermediate objective of this research was to determine whether there are antagonistic actions between IL-27, TER, and ATRA on Treg and Tr1 cells with respect to the evaluated parameters. However, it should be raised here that a solution to the above research problem required the determination of effects on these parameters caused by IL-27, TER, and ATRA when applied alone. Thus, another outcome of the study reported here is gaining better knowledge on the influence of the above agents on Treg and Tr1 cells.

## 2. Results

### 2.1. TER and ATRA Deplete CD4^+^ T Cells

**Figure 1:**
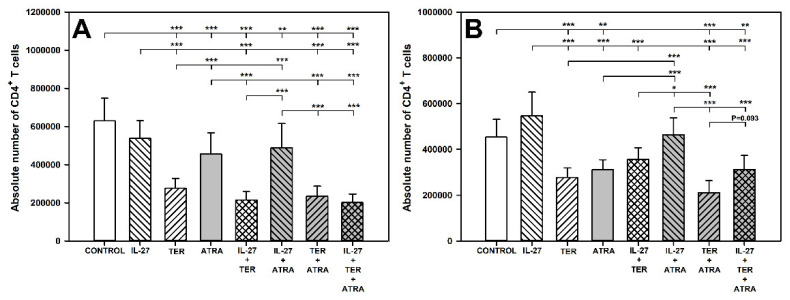
Effect of IL-27, teriflunomide (TER), all-trans-retinoic acid (ATRA), and their combinations on the absolute number of CD4^+^ T cells under stimulated and unstimulated conditions. The parameter was determined in cell cultures incubated in the absence (CONTROL) and presence of IL-27 (100 ng/mL), TER (10^−4^ M), and ATRA (10^−6^ M) for 72 h including concomitant stimulation (with IL-2, anti-CD3, and anti-CD28 and re-stimulation with PMA and ionomycin for the last 5 h before harvest) (**A**) and for 96 h without stimulation (**B**). The absolute count represents the number of CD4^+^ T cells per sample well. Results are expressed as the mean (±S.D.) of three independent experiments with four mice per experiment (overall *n* = 12 (**A**) or *n* = 8 (**B**), * *p* < 0.05, ** *p* < 0.01, *** *p* < 0.001).

The research demonstrated that exposure of cells to TER alone and ATRA alone, but not IL-27 alone, caused a significant reduction in the absolute count of CD4^+^ T cells, both under stimulated and unstimulated conditions (Figure 1A,B). TER-induced depletion of CD4^+^ T cells under stimulated conditions was significantly more profound than that induced by ATRA (Figure 1A). The results indicate that IL-27 and ATRA did not affect the TER-induced depletion of CD4^+^ T cells. The co-exposure of cells to IL-27 + ATRA under unstimulated conditions did not affect the absolute count of CD4^+^ T cells, and the value of this parameter was significantly higher compared to ATRA alone (Figure 1B). It proves that IL-27 antagonized the depleting effect of ATRA on CD4^+^ T cells.

### 2.2. TER and ATRA, but Not IL-27, Reduce the Number of Proliferating Treg and Teff Cells

**Figure 2:**
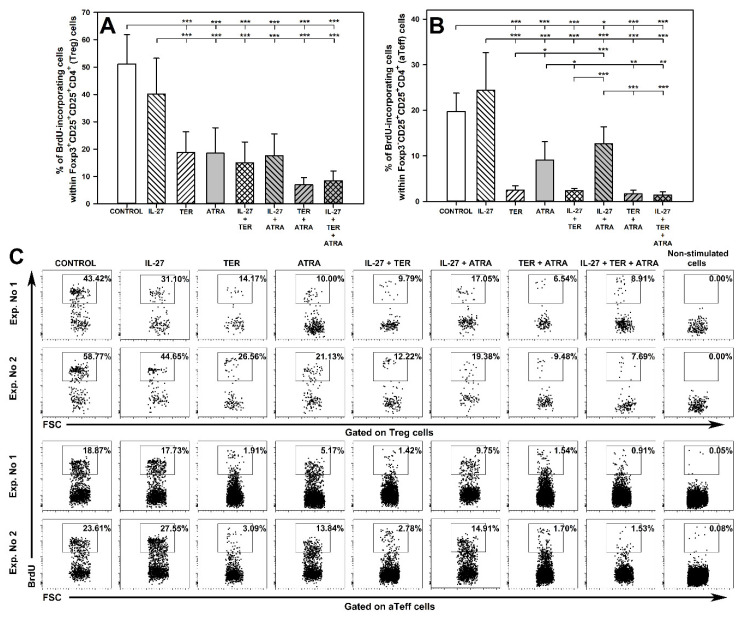
Effect of IL-27, teriflunomide (TER), all-trans-retinoic acid (ATRA), and their combinations on the proliferation of Foxp3^+^CD25^+^CD4^+^ and Foxp3^−^CD25^+^CD4^+^ T cells, i.e., regulatory (Treg) and activated effector (aTeff) T cells, respectively. The relative count of 5-bromo-2-deoxyuridine(BrdU)-incorporating cells within Foxp3^+^CD25^+^CD4^+^ (**A**) and Foxp3^−^CD25^+^CD4^+^ (**B**) T cell subsets was determined in cell cultures incubated in the absence (CONTROL) and presence of IL-27 (100 ng/mL), TER (10^−4^ M), and ATRA (10^−6^ M) for 72 h including concomitant stimulation with IL-2, anti-CD3, and anti-CD28 and re-stimulation with PMA and ionomycin for the last 5 h before harvest. The cells were exposed to BrdU during the last 12 h. Results are expressed as the mean (±S.D.) of two independent experiments with four mice per experiment (overall *n* = 8, * *p* < 0.05, ** *p* < 0.01, *** *p* < 0.001). Examples of dot plot cytograms showing the distribution of BrdU-incorporating cells within Foxp3^+^CD25^+^CD4^+^ (**C**, upper panel) and Foxp3^−^CD25^+^CD4^+^ (**C**, lower panel) T cell subsets.

The exposure of cells to TER alone and ATRA alone and to all the studied combinations considerably decreased the percentage of BrdU^+^Foxp3^+^CD25^+^CD4^+^ (Figure 2A and upper panel of C) and BrdU^+^Foxp3^−^CD25^+^CD4^+^ T cells (Figure 2B and lower panel of C), while IL-27 alone did not alter these parameters. The statistical analysis did not reveal any crucial differences between TER alone, ATRA alone, and all the studied combinations in terms of decreasing the percentage of BrdU^+^Foxp3^+^CD25^+^CD4^+^ T cells (Figure 2A). However, the research demonstrated that the TER-induced reduction in the percentage of BrdU^+^Foxp3^−^CD25^+^CD4^+^ T cells was significantly more profound than that induced by ATRA alone (Figure 2B), and that the co-exposure of cells to IL-27 + TER, TER + ATRA, and IL-27 + TER + ATRA did not affect this parameter. Additionally, the ATRA-induced decrease in the percentage of BrdU^+^Foxp3^−^CD25^+^CD4^+^ T cells did not differ significantly from the value of this parameter obtained in the cultures exposed to the combination of IL-27 + ATRA. Thus, these findings did not provide evidence for the existence of interactions between any treatments with respect to the both discussed parameters (Figure 2A,C).

### 2.3. TER Increases the Percentage and Absolute Count of Foxp3^+^CD25^very high^CD4^+^ T Cells under Activation Conditions

**Figure 3:**
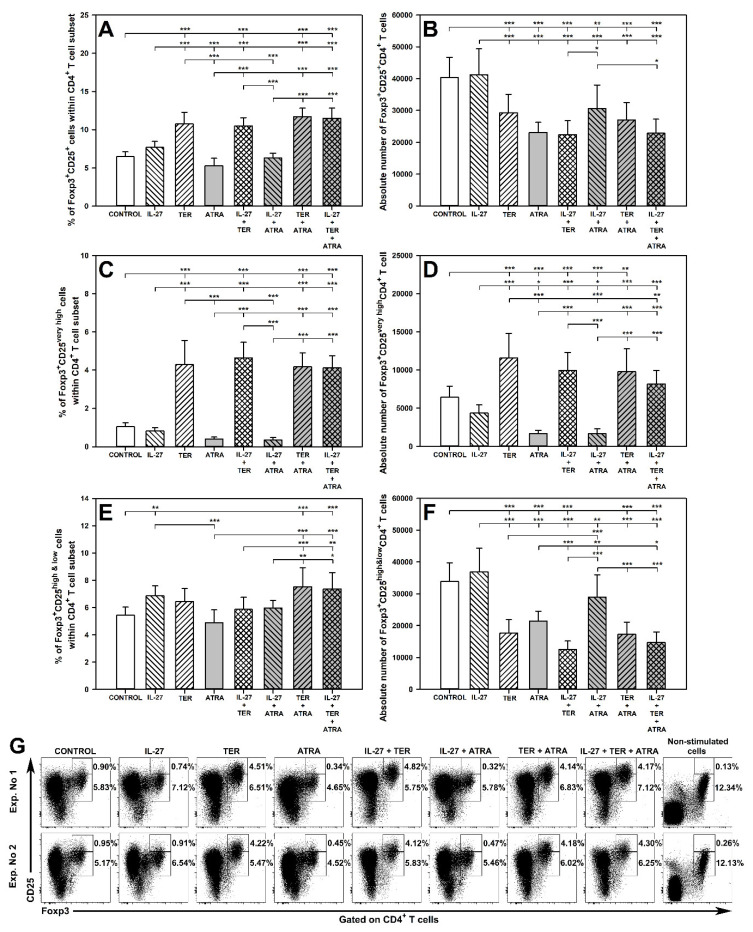
Effect of IL-27, teriflunomide (TER), all-trans-retinoic acid (ATRA), and their combinations on the number of Foxp3-expressing regulatory T (Treg) cells—taking into account their total population and subsets with very high and high + low CD25 expression—under activation conditions. The relative (**A**,**C**,**E**) and absolute count (**B**,**D**,**F**) of particular cell subsets were determined in cell cultures incubated in the absence (CONTROL) and presence of IL-27 (100 ng/mL), TER (10^−4^ M), and ATRA (10^−6^ M) for 72 h including concomitant stimulation with IL-2, anti-CD3, and anti-CD28 and re-stimulation with PMA and ionomycin for the last 5 h before harvest. The relative count is expressed as a percentage of Foxp3^+^CD25^+^ (**A**), Foxp3^+^CD25^very high^ (**C**), and Foxp3^+^CD25^high&low^ (**E**) cells within CD4^+^ T cell subset. The absolute count represents the number of Foxp3^+^CD25^+^CD4^+^ (**B**), Foxp3^+^CD25^very high^ CD4^+^ (**D**), and Foxp3^+^CD25^high&low^CD4^+^ (**F**) T cells per sample well. Results are expressed as the mean (±S.D.) of three independent experiments with four mice per experiment (overall *n* = 12, * *p* < 0.05, ** *p* < 0.01, *** *p* < 0.001). Examples of dot plot cytograms for all treatments show the distribution of Foxp3^+^CD25^+^ cells within CD4^+^ T cell subset (**G**).

Treatment with TER alone led to a significant increase in the percentage of Foxp3^+^CD25^+^CD4^+^ T cells (Figure 3A,G) and their subset with very high CD25 expression (Figure 3C,G), but did not affect the percentage of Foxp3^+^CD25^high&low^CD4^+^ T cells (Figure 3E,G). TER-induced increase in the percentage of Foxp3^+^CD25^very high^CD4^+^ T cells was strongly expressed as the number of these cells was over four times greater than the corresponding control values. Exposure to IL-27 alone did not affect this parameter and the percentage of total Treg subset, i.e., Foxp3^+^CD25^+^CD4^+^ T cells (Figure 3A,C,G), but increased the percentage of Foxp3^+^CD25^high&low^CD4^+^ T cells, although this effect was weakly expressed (Figure 3E,G). The above parameters were not significantly influenced by ATRA alone (Figure 3A,C,E,G).

The exposure of cells to TER alone considerably increased the absolute number of Foxp3^+^CD25^very high^CD4^+^ T cells (Figure 2D), but decreased the absolute number of total Treg cells (Figure 3B) and their Foxp3^+^CD25^high&low^CD4^+^ subset (Figure 3F), while IL-27 alone did not affect these parameters (Figure 3B,D,F). The same findings as for TER alone were observed in the culture co-treated with TER and IL-27, which means that IL-27 did not interact with TER as regards its impact on the above parameters.

The study demonstrated a significant reduction in the absolute count of Foxp3^+^CD25^+^CD4^+^ and Foxp3^+^CD25^very high^CD4^+^ T cells in the samples from the culture exposed to ATRA alone and to the combination of ATRA and IL-27, and the values of these parameters did not differ between both treatments (Figure 3B,D,F). Thus, also in this case, IL-27 did not interact with ATRA-induced effects. However, IL-27 antagonized ATRA-induced decrease in the absolute count of Foxp3^+^CD25^high&low^CD4^+^ T cells. This is evidenced by the absolute count of these cells from samples treated with IL-27 + ATRA being significantly higher than that from samples treated with ATRA alone, and not different from the control values (Figure 3F).

No evidence of interactions between TER and ATRA with respect to the percentage and absolute count of Foxp3^+^CD25^+^CD4^+^ and Foxp3^+^CD25^high&low^CD4^+^ T cells was found (Figure 3B,F). However, it was observed that co-exposure to TER and ATRA led to a significant increase in the absolute count of Foxp3^+^CD25^very high^CD4^+^ T cells, and obviously the value of this parameter was greater than that obtained for the culture treated with ATRA alone (Figure 3D). Therefore, it can be stated that TER fully antagonized ATRA-induced decrease in the absolute count of Foxp3^+^CD25^very high^CD4^+^ T cells. Interestingly, the combination of IL-27 and ATRA abolished TER-induced an increase in the absolute count of Foxp3^+^CD25^very high^CD4^+^ T cells (Figure 3D). The results did not provide the evidence for existence of interactions between the triple combination and dual combinations or single agents with respect to the percentage and absolute count of Foxp3^+^CD25^+^CD4^+^, Foxp3^+^CD25^very high^CD4^+^, and Foxp3^+^CD25^high&low^CD4^+^ T cells (Figure 3).

### 2.4. IL-27 and ATRA Exert a Synergistic Effect on Induction of CD39 Expression on Treg Cells under Activation Conditions

**Figure 4:**
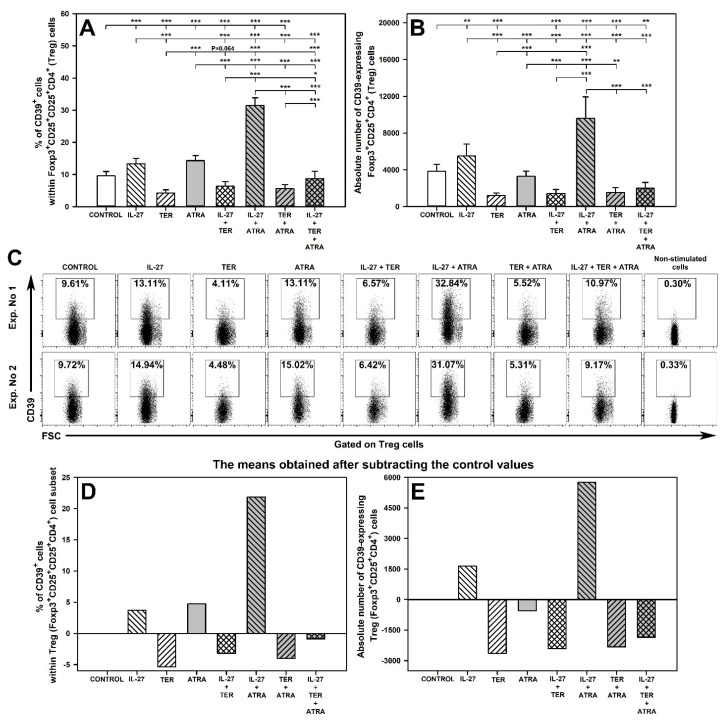
Effect of IL-27, teriflunomide (TER), all-trans-retinoic acid (ATRA), and their combinations on the number of CD39-expressing Foxp3^+^CD25^+^CD4^+^ regulatory T (Treg) cells under activation conditions. The relative (**A**) and absolute count (**B**) of these cells were determined in cell cultures incubated in the absence (CONTROL) and presence of IL-27 (100 ng/mL), TER (10^−4^ M), and ATRA (10^−6^ M) for 72 h including concomitant stimulation with IL-2, anti-CD3, and anti-CD28 and re-stimulation with PMA and ionomycin for the last 5 h before harvest. Unstimulated cells served as a negative control for the cell activation. The relative count is expressed as a percentage of CD39-expressing cells within Foxp3^+^CD25^+^CD4^+^ T cell subset (**A**). The absolute count represents the number of CD39^+^Foxp3^+^CD25^+^CD4^+^ T cells per sample well (**B**). Results are expressed as the mean (±S.D.) of three independent experiments with 4 mice per experiment (overall *n* = 12, * *p* < 0.05, ** *p* < 0.01, *** *p* < 0.001). Examples of dot plot cytograms for all treatments show the distribution of CD39-expressing cells within Foxp3^+^CD25^+^CD4^+^ T cell subset (**C**). Additionally, in order to better visualize the superadditive synergism between IL-27 and ATRA in the induction of CD39 expression on Treg cells, the mean of the relative (**D**) and absolute count (**E**) of studied cells was showed after subtracting the control values.

The research demonstrated that exposure of cells to IL-27 alone and ATRA alone increased the percentage of CD39-expressing cells within Foxp3^+^CD25^+^CD4^+^ T cell subset (Figure 4A,C). Moreover, IL-27 alone induced a significant increase in the absolute count of CD39^+^Foxp3^+^CD25^+^CD4^+^ T cells, while ATRA alone did not alter this parameter (Figure 4B). After subtracting the control values, the mean percentage of CD39-expressing Treg cells induced by exposure to IL-27 alone or ATRA alone was 3.72 and 4.73, respectively, and the mean absolute count of CD39^+^Foxp3^+^CD25^+^CD4^+^ T cells was 1647 and -555 cells, respectively (Figure 4D; Supplementary Material Table S1). Co-exposure to IL-27 and ATRA induced a considerably greater increase in the percentage of CD39-expressing Treg cells compared to that produced by either IL-27 alone or ATRA alone. This effect was spectacular because after the subtraction of control values the mean percentage and absolute count of CD39-expressing Treg cells were found to be 21.88 and 5758, respectively (Figure 4D; Supplementary Material Table S1). These values were significantly greater than those calculated by summing up the values of corresponding parameters obtained in the cultures treated with IL-27 alone or ATRA alone (i.e., 8.45% and 1093, respectively; Supplementary Material Table S1). Thus, the combined effect of IL-27 and ATRA was greater than the sum of the individual effects of these agents alone. These results prove the presence of a superadditive effect between both agents with respect to the induction of CD39 expression on Treg cells under activation conditions. Additionally, taking into consideration the definitions of subtypes of superadditive effect, these findings indicate the occurrence of a synergistic effect between IL-27 and ATRA with respect to increasing the percentage of CD39-expressing Foxp3^+^CD25^+^CD4^+^ T cells, and the enhancement with respect to increasing the absolute number of these cells (Supplementary Material Table S1).

On the contrary, treatment with TER alone significantly reduced the percentage (Figure 4A,C,D) and absolute count (Figure 4B,E) of CD39-expressing Foxp3^+^CD25^+^CD4^+^ T cells. The exposure of cells to the combinations of IL-27 + TER and ATRA + TER resulted in a significant decrease of these parameters compared to control values. Moreover, the percentage of CD39-expressing Foxp3^+^CD25^+^CD4^+^ T cells obtained in the cultures treated with IL-27 + TER and ATRA + TER was lower than that in the cultures treated with IL-27 alone and ATRA alone, respectively, and did not differ considerably from the values of the corresponding parameter obtained in the culture treated with TER alone (Figure 4A). Similarly, the percentage of CD39-expressing Foxp3^+^CD25^+^CD4^+^ T cells obtained in the culture exposed to the combination of IL-27 + TER + ATRA was considerably lower than the values of the corresponding parameter obtained in the culture treated with IL-27 + ATRA, and did not differ from the control values alone (Figure 4A,C,D). Thus, the increase in the percentage and absolute count of CD39-expressing Foxp3^+^CD25^+^CD4^+^ T induced by IL-27 alone, ATRA alone and their combination was abolished by TER. Therefore, the results indicate that under activation conditions, TER fully antagonized the IL-27-induced and ATRA-induced induction of CD39 expression on Treg cells as well as the synergistic effect exerted by both in this respect.

The absolute count of CD39-expressing Foxp3^+^CD25^+^CD4^+^ T cells in the cultures co-treated with IL-27 and TER decreased compared to control values and to the values of this parameter obtained in samples from the culture exposed to IL-27 alone (Figure 4B). Similarly, the absolute count of CD39-expressing Foxp3^+^CD25^+^CD4^+^ T cells exposed to the combination of IL-27 + TER + ATRA was considerably lower than that in the control culture or the culture treated with IL-27 + ATRA (Figure 4B). Thus, TER abrogated the IL-27-induced and IL-27 + ATRA-induced increase in the absolute count of these cells. Therefore, these findings indicate that under activation conditions TER antagonized the IL-27-induced increase in the absolute count of CD39-expressing Foxp3^+^CD25^+^CD4^+^ T cells as well as the enhancement effect exerted by the combination of IL-27 and ATRA in this respect.

### 2.5. Under Unstimulated Conditions IL-27 Decreases and TER Increases the Percentage of Treg Cells and IL-27 Increases the Percentage and Absolute Count of CD39-Expressing Treg Cells

**Figure 5:**
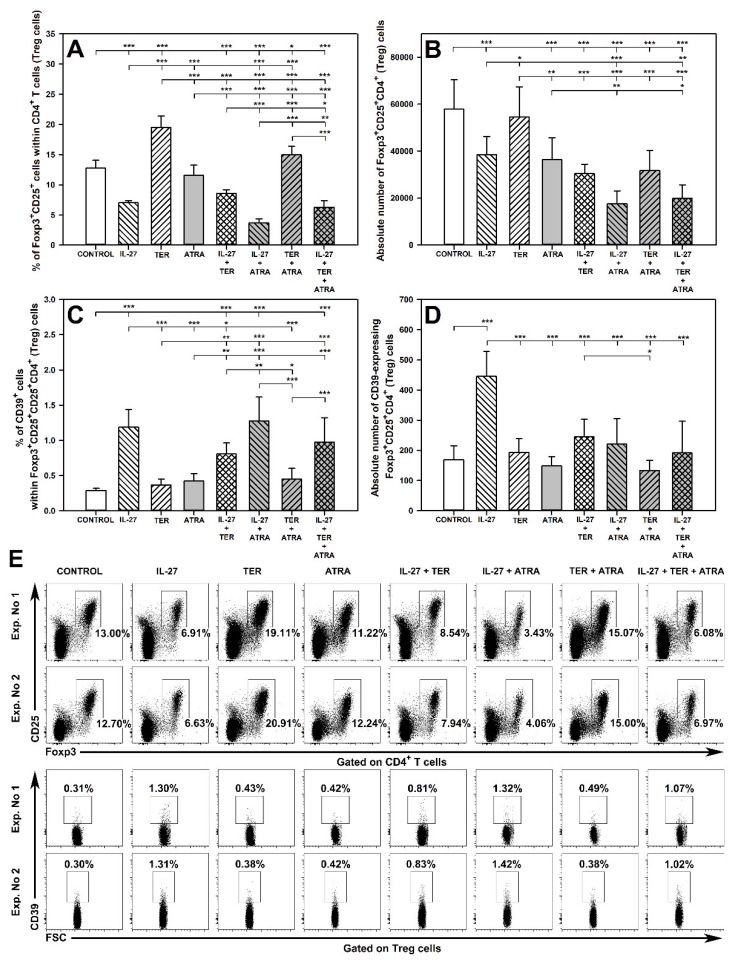
Effect of IL-27, teriflunomide (TER), all-trans-retinoic acid (ATRA), and their combinations on the number of Foxp3^+^CD25^+^CD4^+^ regulatory T (Treg) cells and their CD39-expressing cell subsets under unstimulated conditions. The relative (**A**,**C**) and absolute count (**B**,**D**) of these cells were determined in cell cultures incubated in the absence (CONTROL) and presence of IL-27 (100 ng/mL), TER (10^−4^ M), and ATRA (10^−6^ M) for 96 h. The relative count is expressed as a percentage of Foxp3^+^CD25^+^ cells within CD4^+^ T cell subset (**A**) and CD39^+^ cells within Foxp3^+^CD25^+^CD4^+^ (**C**) T cell subset. The absolute count represents the number of Foxp3^+^CD25^+^CD4^+^ (**B**) and CD39^+^Foxp3^+^CD25^+^CD4^+^ T cells (**D**) per sample well. Results are expressed as the mean (±S.D.) of two independent experiments with four mice per experiment (overall *n* = 8, * *p* < 0.05, ** *p* < 0.01, *** *p* < 0.001). Examples of dot plot cytograms for all treatments show the distribution of Foxp3^+^CD25^+^ (**E**, upper panel) and CD39^+^ (**E**, lower panel) cells within CD4^+^ and Foxp3^+^CD25^+^CD4^+^ T cell subsets, respectively.

Treatment with IL-27 alone significantly reduced the percentage and absolute number of Foxp3^+^CD25^+^CD4^+^ T cells (Figure 5A,B). The exposure of cells to TER alone considerably increased the percentage of Foxp3^+^CD25^+^CD4^+^ T cells, but did not affect the absolute number of these cells (Figure 5A,B). On the contrary, the exposure to ATRA alone caused a significant reduction in the absolute number, but not in the percentage, of these cells (Figure 5A,B).

IL-27 abolished the TER-induced increase in the percentage of Foxp3^+^CD25^+^CD4^+^ T cells. This is proven by the fact that the value of this parameter in samples from the culture treated with the combination of IL-27 and TER was lower compared not only to the culture treated with TER alone, but also to the control values (Figure 5A). Therefore, the results indicate that under unstimulated conditions, IL-27 fully antagonized the TER-induced increase in the percentage of Foxp3^+^CD25^+^CD4^+^ T cells. ATRA also antagonized this TER-induced effect, but only partially. This conclusion is based on the following observation: the percentage of Foxp3^+^CD25^+^CD4^+^ T cells in the cultures co-treated with TER and ATRA was significantly lower than that in the culture treated with TER alone, but it was still greater than the control values (Figure 5A). The combination of IL-27 and ATRA induced significantly greater reduction in the percentage of Foxp3^+^CD25^+^CD4^+^ T cells compared to IL-27 alone (Figure 5A). Such results indicate that ATRA enhanced the IL-27-induced reduction in the percentage of Foxp3^+^CD25^+^CD4^+^ T cells. The percentage of Foxp3^+^CD25^+^CD4^+^ T cells in the culture treated with the triple combination was considerably lower than in the cultures exposed to the combinations of IL-27 + TER and TER + ATRA, but higher than in the culture treated with the combination of IL-27 and ATRA. These results indicate the following interactions: (a) enhancement by ATRA of the antagonistic effect of the IL-27 on TER-induced increase in the percentage of Foxp3^+^CD25^+^CD4^+^ T cells; (b) antagonism by TER of the IL-27 + ATRA-induced enhancement of the reduction in the percentage of Foxp3^+^CD25^+^CD4^+^ T cells.

The absolute number of Foxp3^+^CD25^+^CD4^+^ T cells in the cultures treated with the combinations of IL-27 + TER and TER + ATRA did not differ significantly from the value of this parameter obtained in the cultures exposed to IL-27 alone and ATRA alone (Figure 5B). These results indicate TER did not interact with IL-27 and ATRA with respect to their reducing effect on the absolute number of Foxp3^+^CD25^+^CD4^+^ T cells. The study demonstrated that the combined effect of IL-27 + ATRA on the absolute number of Foxp3^+^CD25^+^CD4^+^ T cells was almost equal to the sum of their individual effects (Figure 5B; Supplementary Material Table S1). Thus, these results indicate an additive interaction between IL-27 and ATRA with respect to their depletive effect on Foxp3^+^CD25^+^CD4^+^ T cells under unstimulated conditions.

The research demonstrated that under unstimulated conditions, exposure of cells to IL-27 alone increased the percentage and absolute number of CD39-expressing Foxp3^+^CD25^+^CD4^+^ T cells, while TER and ATRA did not affect these parameters (Figure 5C,D). The percentage of CD39-expressing Foxp3^+^CD25^+^CD4^+^ T cells in the cultures treated with the combination of IL-27 and TER was significantly lower than that in the culture treated with IL-27 alone, but it was still higher than the control values (Figure 5C). Thus, these results indicate that TER antagonized, but only partially, the IL-27-induced induction of CD39 expression on Foxp3^+^CD25^+^CD4^+^ T cells. ATRA did not interact with this IL-27-induced effect (Figure 5C). In turn, TER and ATRA fully antagonized the IL-27-induced increase in the absolute number of CD39-expressing Foxp3^+^CD25^+^CD4^+^ T cells as the values of this parameter in the culture treated with IL-27 + TER and IL-27 + ATRA were considerably lower than those in the cultures exposed to IL-27 alone and did not differ from the control values (Figure 5D).

The results did not provide the evidence for the existence of interactions between the triple combination and dual combinations or single agents with respect to the absolute number of Foxp3^+^CD25^+^CD4^+^ T cells and the percentage and absolute number of CD39-expressing Foxp3^+^CD25^+^CD4^+^ T cells (Figure 5B–D).

### 2.6. IL-27- and ATRA-Induced Superadditive Effect on the Induction of CD39 Expression on Treg Cells Is Dose-Dependent

As discussed in point 2.4, the combination of IL-27 and ATRA exerted a spectacular synergistic effect on the induction of CD39 expression on Treg cells. Taking into consideration potential clinical implications of the above finding, it seemed pertinent to establish whether this effect was clearly dose-dependent and whether both agents made the same contribution to its induction. Therefore, additional experiments including four combinations of IL-27 and ATRA in varying concentrations were performed.

**Figure 6:**
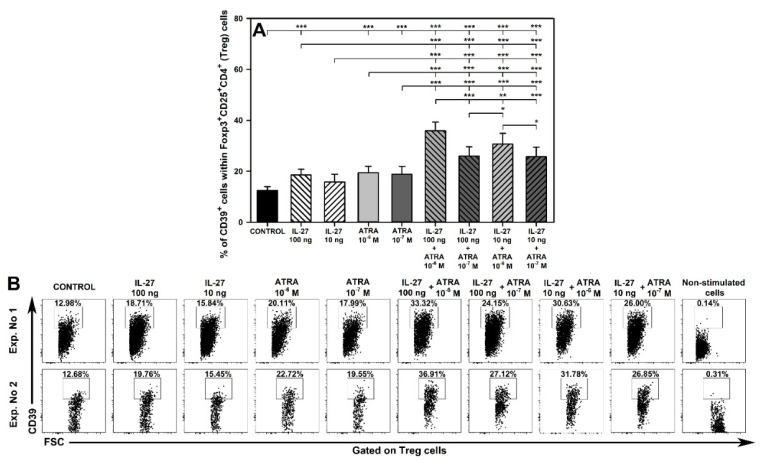
Effect two concentrations of IL-27 and all-trans-retinoic acid (ATRA) and their combinations on the relative count of CD39-expressing Foxp3^+^CD25^+^CD4^+^ regulatory T (Treg) cells under activation conditions (**A**). The parameter was determined in cell cultures incubated in the absence (CONTROL) and presence of IL-27 (100 and 10 ng/mL) and ATRA (10^−6^ and 10^−7^ M) for 72 h including concomitant stimulation with IL-2, anti-CD3, and anti-CD28 and re-stimulation with PMA and ionomycin for the last 5 h before harvest. Unstimulated cells served as a negative control for the cell activation. The relative count is expressed as a percentage of CD39-expressing cells within Foxp3^+^CD25^+^CD4^+^ T cell subset. Results are expressed as the mean (±S.D.) of two independent experiments with five mice per experiment (overall *n* = 10, * *p* < 0.05, ** *p* < 0.01, *** *p* < 0.001). Examples of dot plot cytograms for all treatments show the distribution of CD39-expressing cells within Foxp3^+^CD25^+^CD4^+^ T cell subset (**B**).

The research demonstrated that exposure of cells to IL-27 at 100 ng/mL alone and ATRA at both concentrations alone as well as to all studied combinations of both agents induced a significant increase in the percentage of CD39-expressing cells within Foxp3^+^CD25^+^CD4^+^ T cells (Figure 6). The experiments again confirmed the synergistic action between IL-27 at 100 ng/mL and ATRA 10^−6^ M with regard to the above effect (Figure 6; Supplementary Material Table S2). The combination of IL-27 at 100 ng/mL and ATRA at the lower concentration also exerted a synergistic effect with respect to increasing the percentage of CD39-expressing Foxp3^+^CD25^+^CD4^+^ T cells, although this effect was far less expressed compared to the combination of IL-27 at 100 ng/mL + ATRA 10^−6^ M, and was close to being classified as additive interaction (Figure 6; Supplementary Material Table S2). The combinations of IL-27 at 10 ng/mL with ATRA at both concentrations also induced the superadditive effect on the increase of the discussed parameter (Figure 6A; Supplementary Material Table S2). However, these two interactions should be classified as enhancement rather than synergism because IL-27 at 10 ng/mL alone did not affect the percentage of CD39-expressing Foxp3^+^CD25^+^CD4^+^ T cells (Figure 6). The IL-27 + ATRA combinations ranked as follows according to their statistically significant increasing effect on the percentage of CD39-expressing Foxp3^+^CD25^+^CD4^+^ T cells: IL-27 100 ng/mL + ATRA 10^−6^ M > IL-27 10 ng/mL + ATRA 10^−6^ M > IL-27 100 ng/mL + ATRA 10^−7^ M = IL-27 10 ng/mL + ATRA 10^−7^ M (Figure 6A). Moreover, the mean percentage of CD39-expressing Foxp3^+^CD25^+^CD4^+^ T cells in the cultures treated with IL-27 100 ng/mL + ATRA 10^−7^ M and IL-27 10 ng/mL + ATRA 10^−6^ M constituted 57.61% and 77.56%, respectively, of this parameter obtained in the culture exposed to IL-27 100 ng/mL + ATRA 10^−6^ M (calculated on the basis of data showed in Supplementary Material Table S2). These results indicate that the IL-27- and ATRA-induced superadditive effect on the induction of CD39 expression on Treg cells is dose-dependent. Obviously, both IL-27 and ATRA contribute to the induction of this effect, but the above findings strongly suggest that ATRA constitutes somewhat a more active component of the two.

### 2.7. IL-27 and ATRA in Combination, but Not Alone, Increase the Percentage and Absolute Count of IL-10-Producing Treg Cells and TER Decreases the Percentage and Absolute Count of IL-10-Producing Treg Cells

**Figure 7:**
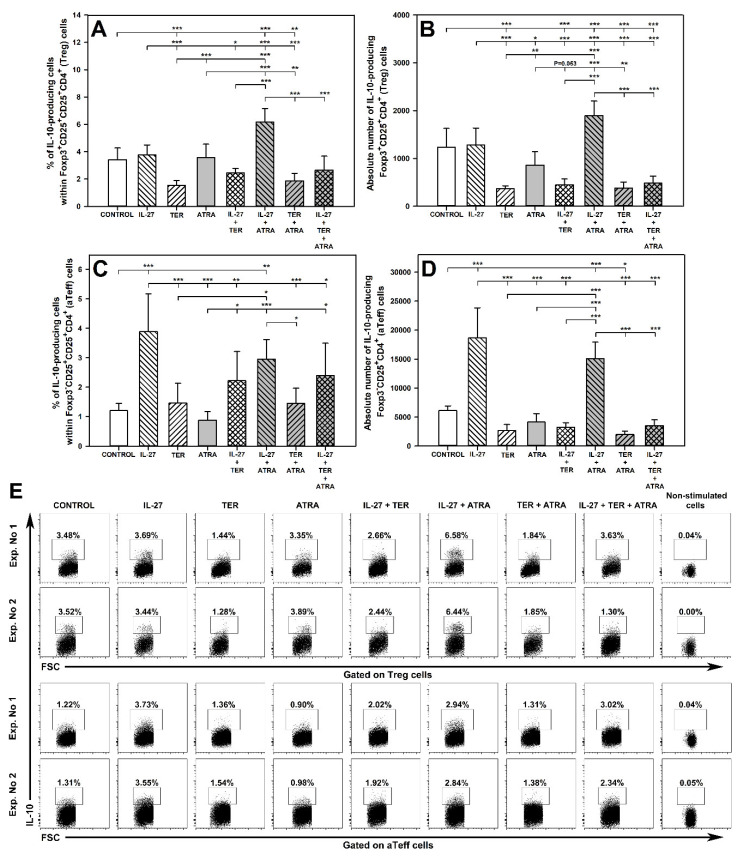
Effect of IL-27, teriflunomide (TER), all-trans-retinoic acid (ATRA), and their combinations on the number of IL-10-producing Foxp3^+^CD25^+^CD4^+^ and Foxp3^−^CD25^+^CD4^+^ T cells, i.e., regulatory (Treg) and activated effector (aTeff) T cells, respectively. The relative (**A**,**C**) and absolute count (**B**,**D**) of particular cell subsets were determined in cell cultures incubated in the absence (CONTROL) and presence of IL-27 (100 ng/mL), TER (10^−4^ M), and ATRA (10^−6^ M) for 72 h including concomitant stimulation with IL-2, anti-CD3, and anti-CD28 and re-stimulation with PMA and ionomycin for the last 5 h before harvest. Unstimulated cells served as a negative control for the cell activation. The relative count is expressed as a percentage of IL-10-producing cells within Foxp3^+^CD25^+^CD4^+^ (**A**) and Foxp3^−^CD25^+^CD4^+^ (**C**) T cell subsets. The absolute count represents the number of IL-10^+^Foxp3^+^CD25^+^CD4^+^ (**B**) and IL-10^+^Foxp3^−^CD25^+^CD4^+^ (**D**) T cells per sample well. Results are expressed as the mean (±S.D.) of two independent experiments with 4 mice per experiment (overall *n* = 8, * *p* < 0.05, ** *p* < 0.01, *** *p* < 0.001). Examples of dot plot cytograms showing the distribution of IL-10-producing cells within Foxp3^+^CD25^+^CD4^+^ (**E**, upper panel) and Foxp3^−^CD25^+^CD4^+^ (**E**, lower panel) T cell subsets.

The exposure of cells to IL-27 alone and ATRA alone did not influence the percentage and absolute number of IL-10-producing Foxp3^+^CD25^+^CD4^+^ T cells (Figure 7A,B and upper panel of E). Interestingly, the exposure of cells to the combination of IL-27 and ATRA significantly increased the value of these parameters (Figure 7A,B and upper panel of E). This is another example of the interaction classified here as ‘induction of an effect’. On the contrary, the treatment with TER alone significantly reduced the percentage and absolute number of IL-10-producing Foxp3^+^CD25^+^CD4^+^ T cells, and co-exposure of cells to TER with IL-27 or ATRA did not influence this effect (Figure 7A,B and upper panel of E). Thus, TER did not interact with IL-27 and ATRA with respect to the above effect. However, the percentage of IL-10-producing Foxp3^+^CD25^+^CD4^+^ T cells in the culture treated with the triple combination was considerably lower than in the culture co-exposed to IL-27 and ATRA, and did not differ from the control values (Figure 7A and upper panel of E). In turn, the exposure of cells to the triple combination caused significant reduction in the absolute number of these cells in comparison with both the IL-27 + ATRA combination and the control (Figure 7B). These results indicate the following interactions: (a) antagonism by TER of IL-27 + ATRA-induced increase in the percentage and absolute number of IL-10-producing Foxp3^+^CD25^+^CD4^+^ T cells; (b) antagonism by the combination of IL-27 + ATRA of TER-induced reduction in the percentage of IL-10-producing Foxp3^+^CD25^+^CD4^+^ T cells.

### 2.8. IL-27 Induces an Increase in the Percentage and Absolute Number of IL-10-Producing aTeff Cells and TER Antagonizes This Effect

The research demonstrated that the treatment with IL-27 alone induced a significant increase in the percentage and absolute number of IL-10-producing Foxp3^−^CD25^+^CD4^+^ T cells, while TER alone and ATRA alone did not influence these parameters (Figure 7C,D and lower panel of E). The co-exposure of cells to IL-27 and ATRA did not affect the IL-27-induced increase in these parameters (Figure 7C,D and lower panel of E). Therefore, it can be stated that ATRA did not interact with IL-27 with regard to its increasing effect on the percentage and absolute number of IL-10-producing Foxp3^−^CD25^+^CD4^+^ T cells. On the contrary, TER fully antagonized the IL-27-induced increase in the percentage and absolute number of IL-10-producing Foxp3^−^CD25^+^CD4^+^ T cells. This is proven by the fact that the percentage and absolute number of these cells in the culture treated with IL-27 + TER were significantly lower than those in the culture exposed to IL-27 alone, and did not differ from the control values (Figure 7C,D and lower panel of E). Although the treatment with TER alone and ATRA alone did not affect the discussed parameters, the exposure of cells to their combination induced a reduction in the absolute number of IL-10-producing Foxp3^−^CD25^+^CD4^+^ T cells (Figure 7D). Thus, this effect represents the interaction type classified here as ‘induction of an effect’.

### 2.9. TER Increases the Percentage of TGF-β-Producing Treg Cells

**Figure 8:**
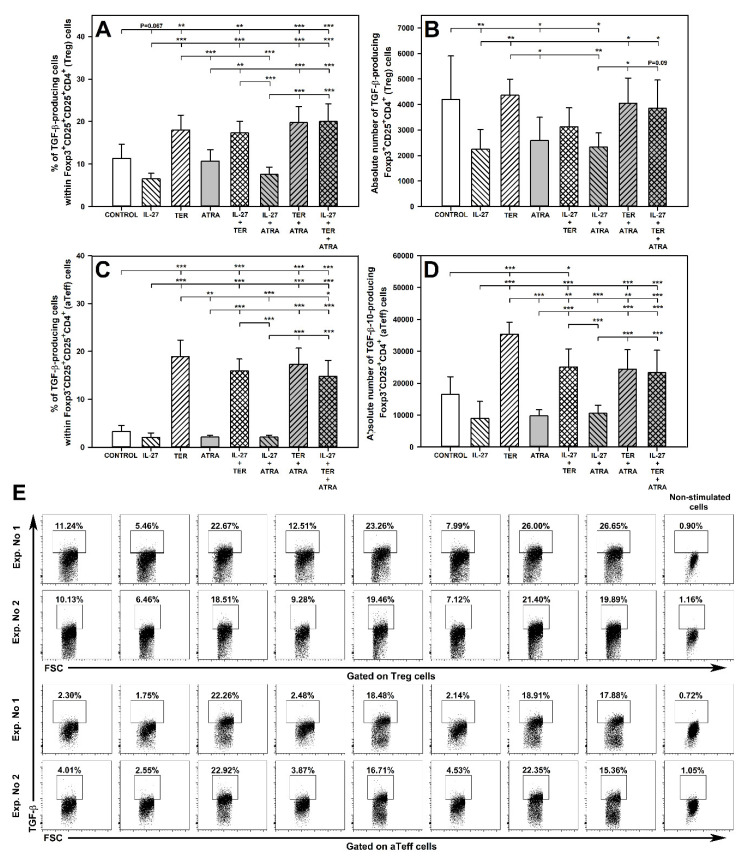
Effect of IL-27, teriflunomide (TER), all-trans-retinoic acid (ATRA), and their combinations on the number of TGF-β-producing Foxp3^+^CD25^+^CD4^+^ and Foxp3^−^CD25^+^CD4^+^ T cells, i.e., regulatory (Treg) and activated effector (aTeff) T cells, respectively. The relative (**A**,**C**) and absolute count (**B**,**D**) of particular cell subsets were determined in cell cultures incubated in the absence (CONTROL) and presence of IL-27 (100 ng/mL), TER (10^−4^ M), and ATRA (10^−6^ M) for 72 h including concomitant stimulation with IL-2, anti-CD3, and anti-CD28 and re-stimulation with PMA and ionomycin for the last 5 h before harvest. Unstimulated cells served as a negative control for the cell activation. The relative count is expressed as a percentage of TGF-β-producing cells within Foxp3^+^CD25^+^CD4^+^ (**A**) and Foxp3^−^CD25^+^CD4^+^ (**C**) T cell subsets. The absolute count represents the number of TGF-β^+^Foxp3^+^CD25^+^CD4^+^ (**B**) and TGF-β^+^Foxp3^−^CD25^+^CD4^+^ (**D**) T cells per sample well. Results are expressed as the mean (±S.D.) of two independent experiments with four mice per experiment (overall *n* = 8, * *p* < 0.05, ** *p* < 0.01, *** *p* < 0.001). Examples of dot plot cytograms showing the distribution of TGF-β-producing cells within Foxp3^+^CD25^+^CD4^+^ (**E**, upper panel) and Foxp3^−^CD25^+^CD4^+^ (**E**, lower panel) T cell subsets.

The exposure of cells to TER alone did not affect the absolute number of TGF-β-producing Foxp3^+^CD25^+^CD4^+^ T cells, but significantly increased the percentage of these cells, whereas the co-exposure of cells to TER with IL-27 or ATRA did not alter this effect (Figure 8A,B and upper panel of E). The results indicate that IL-27 and ATRA did not interact with TER with respect to its increasing action on the percentage of TGF-β-producing Foxp3^+^CD25^+^CD4^+^ T cells. The treatment with IL-27 alone and ATRA alone did not influence the percentage of TGF-β-producing Foxp3^+^CD25^+^CD4^+^ T cells, although a certain trend (*p* = 0.067) toward decreasing this parameter was observed in the culture exposed to IL-27 alone (Figure 8A and upper panel of E). The treatment with IL-27 alone, ATRA alone, and their combination led to a significant reduction in the absolute number of TGF-β-producing Foxp3^+^CD25^+^CD4^+^ T cells, while these effects were absent in the cultures exposed to the combinations of IL-27 + TER, TER + ATRA and IL-27 + TER + ATRA (Figure 8B). Therefore, it can be stated that TER antagonized the depleting effect of IL-27 and ATRA on TGF-β-producing Foxp3^+^CD25^+^CD4^+^ T cells.

### 2.10. TER Induces an Increase in the Percentage and Absolute Number of TGF-β-Producing aTeff Cells

The study demonstrated that the exposure of cells to TER alone led to a significant increase in the percentage of TGF-β-producing Foxp3^−^CD25^+^CD4^+^ T cells, while IL-27 and ATRA did not influence this parameter (Figure 8C and lower panel of E). The TER-induced increase in the percentage of TGF-β-producing Foxp3^−^CD25^+^CD4^+^ T cells represented an exceptionally large-scale effect as the number of these cells was approximately six times greater than the control values (Figure 8C). The percentage of TGF-β-producing Foxp3^−^CD25^+^CD4^+^ T cells in the culture treated with TER alone did not differ from that in the cultures exposed to the combinations of IL-27 + TER and TER + ATRA. Therefore, same as in case of Treg cells, it can be stated that IL-27 and ATRA did not interact with TER with respect to its increasing action on the percentage of TGF-β-producing Foxp3^−^CD25^+^CD4^+^ T cells. Interestingly, the percentage of these cells in the culture exposed to the triple combination was lower than in the culture treated with TER alone, although it was still 4.5-fold higher than the control values (Figure 8C). Thus, it can be concluded that the combination of IL-27 and ATRA antagonized, but to a very limited extent, the TER-induced increase in the TGF-β-producing Foxp3^−^CD25^+^CD4^+^ T cells.

Similarly to the percentage results, the study demonstrated that the treatment with TER alone induced a significant increase in the absolute number of TGF-β-producing Foxp3^−^CD25^+^CD4^+^ T cells, while IL-27 and ATRA did not affect this parameter (Figure 8D). However, IL-27 alone and ATRA alone as well as their combination antagonized the TER-induced increase in the absolute number of TGF-β-producing Foxp3^−^CD25^+^CD4^+^ T cells. This is demonstrated by the fact the values of this parameter in the culture treated with the combinations of IL-27 + TER, TER + ATRA, and IL-27 + TER + ATRA were considerably lower than those in the culture treated with TER alone (Figure 8D). The results indicate that IL-27 and ATRA did not potentiate each other in terms of their effect on the TER-induced increase in the absolute number of TGF-β-producing Foxp3^−^CD25^+^CD4^+^ T cells, as the number of these cells in the culture exposed to the triple combination did not differ from that in the cultures treated with IL-27 alone or ATRA alone (Figure 8D).

### 2.11. TER Decreases the Induction of Tr1 Cells as Well as Abrogates IL-27-Mediated Induction of These Cells

**Figure 9:**
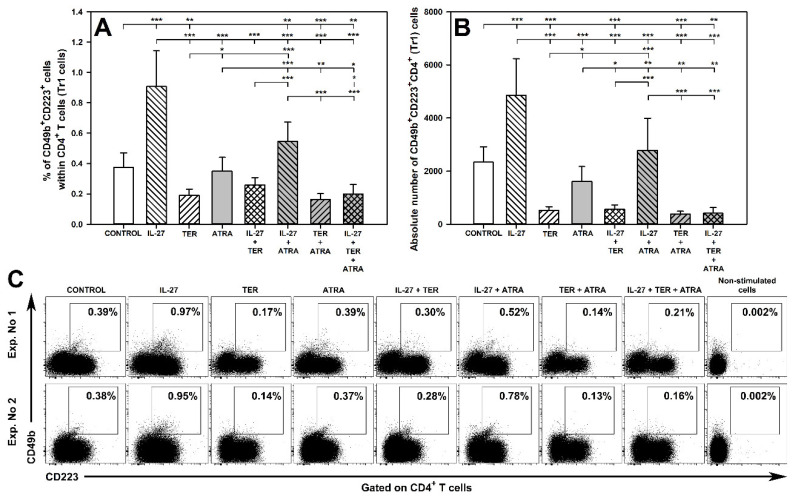
Effect of IL-27, teriflunomide (TER), all-trans-retinoic acid (ATRA), and their combinations on the number of CD49b^+^CD223^+^CD4^+^ T cells, i.e., type 1 regulatory T (Tr1) cells. The relative (**A**) and absolute count (**B**) of these cells were determined in cell cultures incubated in the absence (CONTROL) and presence of IL-27 (100 ng/mL), TER (10^−4^ M), and ATRA (10^−6^ M) for 72 h including concomitant stimulation with IL-2, anti-CD3, and anti-CD28 and re-stimulation with PMA and ionomycin for the last 5 h before harvest. Unstimulated cells served as a negative control for the cell activation. The relative count is expressed as a percentage of CD49b^+^CD223^+^ cells within CD4^+^ T cell subset (**A**). The absolute count represents the number of CD49b^+^CD223^+^CD4^+^ T cells per sample well (**B**). Results are expressed as the mean (±S.D.) of three independent experiments with four mice per experiment (overall *n* = 12, * *p* < 0.05, ** *p* < 0.01, *** *p* < 0.001). Examples of dot plot cytograms for all treatments show the distribution of CD49b^+^CD223^+^ cells within CD4^+^ T cell subset (**C**).

As expected, the treatment with IL-27 alone induced a significant increase in the percentage and absolute number of CD49b^+^CD223^+^CD4^+^ T cells (Figure 9A–C). On the contrary, the exposure of cells to TER alone led to a significant reduction in these parameters, while ATRA alone did not influence them (Figure 9A–C). The percentage and absolute number of CD49b^+^CD223^+^CD4^+^ T cells in the culture co-treated with IL-27 and TER were lower than those in the culture treated with IL-27 alone. What is more, the value of the former parameter did not differ significantly from that in the control and TER alone-treated cultures, while the value of the latter parameter was even lower than the control values (Figure 9A,B). In turn, the percentage of CD49b^+^CD223^+^CD4^+^ T cells in the culture co-treated with IL-27 and ATRA was lower than in the culture exposed to IL-27 alone, but still higher than the control values (Figure 9A); the absolute number of CD49b^+^CD223^+^CD4^+^ T cells in the culture co-exposed to both agents was also lower compared to the culture exposed to IL-27 alone, but did not differ from the control values (Figure 9B). Taking above into consideration, it can be concluded that TER fully antagonized the IL-27-mediated induction of CD49b^+^CD223^+^CD4^+^ T cells, while IL-27 seemingly had no clear influence on the TER-induced decrease in the induction of these cells. ATRA also antagonized the IL-27-mediated induction of CD49b^+^CD223^+^CD4^+^ T cells. However, the percentage data indicate that ATRA only partially abolished this effect (Figure 9A).

## 3. Discussion

The study found that TER alone and ATRA alone, but not IL-27, induced depletion of CD4^+^ T cells, both under stimulated and unstimulated conditions. In unstimulated cultures, it must have been a consequence of increased cell death because lymphocytes practically do not proliferate under such conditions. This assertion is supported by reports suggesting that TER [55] and ATRA [56] may exert the pro-apoptotic effect on T cells. In stimulated cultures (that is, in cultures where intensive cell proliferation occurs), this was additionally due to the antiproliferative action of TER and ATRA. This research showed that both TER alone and ATRA alone exerted the antiproliferative effect on Treg and aTeff cells, with the TER-induced inhibition of proliferation being greater than that induced by ATRA. This is in agreement with the results concerning the effect of these agents on the absolute number of CD4^+^ T cells, as the TER-induced depletion of CD4^+^ T cells under stimulated conditions was significantly more profound than that induced by ATRA. It is actually due to these depletive and antiproliferative actions that the TER- or ATRA-induced increases in the percentage values of some parameters are not reflected by the corresponding increases in the absolute values. Thus, these determinations are highly important for interpretating and understanding properly the results regarding absolute values.

It is widely accepted that the antiproliferative effect of the drug on T and B cells constitutes the main mechanism responsible for the immunomodulatory action of TER [39]. It was demonstrated that the percentage and absolute numbers of lymphocytes were reduced compared with baseline in patients treated with TER, although this decline was considered as not clinically significant [57,58]. Tang et al. [59] found that treatment of mice with ATRA inhibited proliferation of T cells and dramatically decreased their absolute numbers. Moreover, it was demonstrated that ATRA inhibited proliferation of murine lymphoid precursors [60] and bovine peripheral blood mononuclear leukocytes [61]. Therefore, the depletive and antiproliferative effects of TER and ATRA might be expected, especially that such effects of the drugs are more strongly manifested under in vitro conditions compared to in vivo conditions.

The present study demonstrated that TER increased the percentage of Foxp3^+^CD25^+^ cells within CD4^+^ T cell subsets both under stimulated and unstimulated conditions; it should be added that the stimulation used in the study mimics in vivo T cell activation. This finding strongly suggests that TER induces Foxp3 expression in Foxp3-negative CD4^+^ T cells, and thereby induces/promotes conversion of conventional CD4^+^ T cells into iTreg cells. Both the finding and conclusion are in agreement with the studies conducted in our department on the effect of TER on canine T cells [49]. Additionally, the study by Ochoa-Repáraz et al. [33] strongly suggested that TER induced Foxp3 expression in CD4^+^ T cells. Interestingly, the present study demonstrated that these TER-induced Foxp3^+^ cells under stimulated conditions were characterized by very high expression of CD25, implicating that TER simultaneously induced Foxp3 expression and increased the density of CD25 molecules. The increase in the percentage of Foxp3^+^CD25^very high^CD4^+^ T cells was accompanied by an increase in the absolute number of these cells. Although TER increased the percentage of the total Treg subset, it either reduced or left unaltered (depending on the conditions) the absolute counts of these cells, which was most probably linked to the depletive effect of the drug on CD4^+^ T cells. Notwithstanding this, the study revealed that TER treatment shifted the Treg-cell/Teff-cell balance toward Treg cells.

The fact that the TER-induced expression of Foxp3 was accompanied by the up-regulation of the density of CD25 molecules may not be without significance. Since Treg cells constitutively express CD25, the high-affinity receptor for IL-2, one of the earliest mechanisms proposed for Treg cells suppressor activity was that Treg cells suppress by ‘sopping up’ IL-2 produced by Teff cells, thereby preventing their proliferation and differentiation [62]. Although the involvement of this mechanism in Treg cell function was contested [63], several studies strongly suggest that consumption of IL-2 by Treg cells plays as critical a role in restraining the activation and expansion of Teff cells, especially CD8^+^ T cells [64,65,66]. While the present study does not fully substantiate any definitive conclusions regarding the discussed topic, it can be hypothesized that an additional mechanism mediating the immunosuppressive action of TER may consist in the up-regulation of the density of CD25 on Treg cells. In the light of the studies cited above, such action should deprive Teff cells of IL-2 (as a result of an intensified uptake of this cytokine from the microenvironment), which in consequence leads to the inhibition of Teff cell proliferation and activation.

The results indicate that IL-27 had no effect on Foxp3 expression in Treg cells under activation conditions. This is in line with previous studies [67], which also found that IL-27 did not alter Foxp3 expression under stimulation by anti-CD3/28 antibodies. Mice deficient in IL-27 have normal Treg cell development [68,69], suggesting that IL-27 is not necessary for this process. However, under unstimulated conditions, IL-27 decreased the percentage and absolute count of Treg cells but did not affect the absolute count of the entire CD4^+^ T cell subset. It indicates that IL-27 could down-regulate Foxp3 expression in Treg cells. Thus, the results suggest that the effect of IL-27 on Foxp3 expression in already existing Treg cells may depend on their activation status. The study demonstrated that ATRA had no effect on Foxp3 expression in Treg cells, but caused loss of these cells, which was most probably linked to its depletive effect on CD4^+^ T cells. Several studies [52,53,54] demonstrated that ATRA promoted TGF-β-induced differentiation of naive human and murine CD4^+^ T cells into Treg cells. The literature lacks any study on the effect of ATRA on Foxp3 expression and/or the number of Treg cells without concomitant TGF-β treatment. Taking into account the results obtained here, it can be concluded that IL-27 alone and ATRA alone do not induce the generation of iTreg cells.

The results of the study indicate that IL-27 alone and ATRA alone up-regulated, but TER down-regulated, the activation-induced CD39 expression on Treg cells. Moreover, IL-27 increased the constitutive expression of CD39 these cells, while TER and ATRA did not affect it. These findings suggest that IL-27 and ATRA may enhance, while TER may impair, the suppressive activity of Treg cells in the aspect of their capacity to express CD39. To the author’s best knowledge, only one study reported that IL-27 induced CD39 expression on Treg cells [70]. Additionally, there are hardly any studies addressing the effect of TER on the discussed topic. Nevertheless, it was reported that the treatment with TER of patients with multiple sclerosis did not affect the expression of CD39 on peripheral blood Treg cells [71]. However, the result was evaluated only after a year of treatment with TER, hence the effect of the drug might not have been captured.

The research demonstrated that the treatment with IL-27 alone did not affect the number of IL-10-producing Treg cells, but induced the ability of aTeff cells to produce this cytokine. This applies most likely to Th2 cells but also to Tr1 cells, because they are primary producers of IL-10 within the non-Treg CD4^+^ T cell subset (hence qualifying Foxp3^−^CD25^+^CD4^+^ T cells in this paper as aTeff cells is a kind of simplification, because this subset also include Tr1 cells). This finding is in full compliance with the results of Batten et al. [48], who demonstrated that the IL-27 treatment induced IL-10-producion within the Foxp3-negative, but not Foxp3-positive, CD4^+^ T cell subset. On the contrary, TER alone suppressed the capacity of Treg cells to produce IL-10 but did not affect aTeff cells in this respect. The results indicate that IL-27 did not increase the capacity of Treg cells to produce TGF-β, but instead exhibited a trend toward decreasing this capacity. In turn, TER triggered TGF-β production in Treg and Teff cells, and this effect was specially strongly expressed with regard to aTeff cells. This conclusion is in agreement with the results by Cao et al. [51] who stated that augmentation of TGF-β production is an additional mechanism underlying the immunomodulatory effect of TER.

As it could be expected from previous reports [45,46,47], the treatment with IL-27 induced the generation of Tr1 cells. On the contrary, TER was found to counteract the induction of these cells. However, in the only available study on this topic [50], it was found that teriflunomide increased the percentage of Tr1 cells in blood, although not in the spleen, of mice with experimental autoimmune encephalomyelitis. The study demonstrated that ATRA did not change the number of Tr1 cells or the number of IL-10- and TGF-β-producing Treg and aTeff cells, which indicates that it has no influence on the ability of Treg cells to produce these cytokines. The literature lacks studies on the effect of ATRA on Tr1 cells and IL-10 and TGF-β production by CD4^+^ T cells.

Whenever referring to a desired or undesired effect or interaction in this paper, the author means effects and interactions that are desired or undesired from the point of view of the purpose of this research. Should one wish to analyze the results of this study in terms of weakening the action of Treg cells, then the terms ‘desired’ and ‘undesired’ would be applied conversely. To recapitulate the desired effects produced by each analyzed agent, the following needs to be brought to our attention:

- IL-27: (a) induced CD39 expression on Treg cells; (b) induced the ability of aTeff cells to IL-10 production; (c) induced the generation of Tr1 cells.
- TER: (a) induced Foxp3-expressing CD4^+^ T cells characterized by more dense expression of CD25; (b) induced the ability of Treg and aTeff cells to TGF-β production.
- ATRA: induced CD39 expression on Treg cells.
- In turn, the undesired effects of each substance can be summarized as follows:
- IL-27: down-regulated Foxp3 expression on Treg cells under unstimulated conditions.
- TER: (a) down-regulated CD39 expression on Treg cells; (b) suppressed the capacity of Treg cells to produce IL-10; (c) counteracted the induction of Tr1 cells.

The principal scientific problem undertaken in this research has been to make an attempt at inducing pharmacologically iTreg cells that will be additionally characterized by a highly suppressive phenotype through the combined treatment with IL-27, teriflunomide, and ATRA. Considering the effects produced by each agent, their combination theoretically should induce the generation of the Foxp3^+^CD25^very high^CD4^+^ T cell subset characterized by increased TGF-β production (the effects induced by TER) and CD39 expression (the effect induced by IL-27 and ATRA). Although none of these agents affected the number of IL-10-producing Treg cells, IL-27 induced the ability of remaining CD4^+^ T cells to produce IL-10; IL-10 produced by these cells exerted similar effects (i.e., anti-inflammatory and immunosuppressive) to those produced by Treg cells. However, the combination of IL-27, TER, and ATRA did not induce the generation of a cell subset with all aforementioned attributes. Due to the antagonism between these agents, certain effects were either abolished or reduced. In turn, such interactions as synergism, enhancement, or ‘induction of an effect’, either enhanced or induced some desired effects. Two desired interactions were determined; they appeared between IL-27 and ATRA, and consisted in: (a) a spectacular synergistic effect between IL-27 and ATRA with respect to increasing CD39 expression on Treg cells under activation conditions; (b) induction of the ability of Treg cells to produce IL-10. Two undesired interactions were noted between IL-27 and ATRA: (a) ATRA enhanced IL-27-induced down-regulation of Foxp3 expression on Treg cells but only under unstimulated conditions, while these agents (alone and in combination) did not affect it under activation conditions; (b) ATRA antagonized of IL-27-induced Tr1 cell generation.

Thus, the results strongly suggest that in T cell activation conditions, co-exposure of lymphocytes to IL-27 and ATRA will not result in the induction of iTreg cells, but may significantly enhance the suppressive function of existing Treg cells via up-regulation of CD39 expression and IL-10 production, i.e., two crucial immunosuppressive molecules responsible for this function. It may not necessarily be associated with combined administration of IL-27 and ATRA to patients because IL-27 occurs in the body naturally. In a recent study, it has been determined that the mean concentration of IL-27 in the serum of healthy volunteers was about 6 ng/mL [72]. The present study demonstrated that the combinations of IL-27 at 10 ng/mL with ATRA induced the superadditive effect on the increase in the number of CD39-expressing Treg cells. This suggests that such an interaction can be present in the body as a consequence of the administration of ATRA alone.

The research demonstrated that TER antagonized or more specifically abolished all the previously listed desired effects induced by IL-27 alone, ATRA alone and their combination. In turn, IL-27 alone, ATRA alone, and their combination affected the TER induced desired effects to a lesser extent; some of them were diminished, but in most cases they were unaffected. Thus, it can be concluded that the triple combination treatment with TER, IL-27 and ATRA or TER and ATRA (assuming the involvement of endogenous IL-27 in the interaction with TER and ATRA) does not provide any benefits over TER alone or the dual combination including IL-27 and ATRA in terms of the pharmacological induction of iTreg cells with a highly suppressive phenotype. What is more, the mentioned triple combination treatment may be less effective due to mutual antagonistic interactions between TER and IL-27 + ATRA. In the author’s opinion, however, these antagonistic interactions do not rule out using the desired effects induced by TER and the combination of IL-27 and ATRA for generation of iTreg cells with a highly suppressive phenotype. The research concept should only be modified. The concept applied in this study was to generate ‘Super Tregs’ by means of a combination treatment (i.e., simultaneous exposure to IL-27, TER, and ATRA), which, however, resulted in certain undesired interactions. Instead, a sequential treatment should be considered, composed of the initial use of TER to induce Foxp3-expressing CD25^very high^ CD4^+^ T cells and to trigger the ability of Treg to TGF-β production, followed by treatment with ATRA and IL-27 (after discontinuation of TER) in order to induce CD39 expression and IL-10 production in existing and TER-induced Treg cells. Such an approach could allow one to avoid the aforementioned undesired interactions and to achieve the principal scientific goal of this study.

## 4. Materials and Methods

### 4.1. Animals

The experiments were carried out on female 8-week-old Balb/c mice. Mice were bred and maintained under standard laboratory conditions (12/12 h light cycle, controlled temperature (21 ± 2 °C) and humidity (55 ± 5%)) with ad libitum access to food and water, in the Animal Facility of the Faculty of Veterinary Medicine, University of Warmia and Mazury in Olsztyn. Mice were euthanized by asphyxiation with CO2. Law in Poland (Act of 15 January 2015 on the Protection of Animals Used for Scientific or Educational Purposes) does not require a permit from an ethics commission to conduct experiments in which samples for research are obtained post mortem from animals not submitted to any procedure while alive.

### 4.2. Isolation of Lymphocytes

Submandibular gland, parotid gland, deep cervical, axillary, and mesenteric lymph nodes were harvested and subjected to Dounce homogenization. The resulting cell suspensions were filtered through a 70 μm cell strainer (BD Biosciences, East Rutherford, NJ, USA) and washed (300× *g* for 5 min at 5 °C; the same parameters were used for all cell-washing procedures) and re-suspended in complete medium (CM; RPMI 1640, 10% FBS, 10 mM HEPES buffer, 10 mM nonessential amino acids, 10 mM sodium pyruvate, and 10 U/mL penicillin/streptomycin (all from Sigma-Aldrich, Munich, Germany)).

### 4.3. In Vitro Stimulation and Culture Conditions

The cells were adjusted to a final concentration of 4 × 10^6^ cells/mL in CM and seeded in 24-well plates in 1 mL aliquots and incubated for 72 (experiments performed under stimulation conditions) and 96 h (experiments performed under unstimulated conditions) in the absence (CONTROL) or presence of IL-27 (Recombinant mouse IL-27; BioLegend, San Diego, CA, USA), TER, and ATRA (both from Tocris Bioscience, Bristol, UK) and their combinations. In each experiment, cells were exposed to TER and ATRA in concentrations reflecting their plasma levels achievable in vivo, i.e., 10^−4^ [73] and 10^−6^ M [74,75], respectively. As regards IL-27, its concentration (i.e., 100 ng/mL) was chosen on the basis of relevant published reports [76,77] and preliminary experiments. Additionally, in comparative experiments on the effect of various concentration combinations of IL-27 and ATRA on CD39 expression on Treg cells (Figure 5), ten-fold lower concentrations of both agents were used. TER and ATRA were dissolved in DMSO; therefore, the same amount of DMSO was added to control and IL-27 treated cells. IL-27 was diluted in 1% bovine serum albumin (BSA; carrier protein; Sigma-Aldrich) in phosphate buffered saline (PBS; Dulbecco’s PBS devoid of Ca^2+^ and Mg^2+^; Sigma-Aldrich); therefore, the same amount of % BSA in PBS was added to control and TER- and ATRA-treated cells. Cells were stimulated similarly as in our previous works [78,79] and others’ works [80,81,82]. Briefly, cells were activated for 72 h with plate-coated anti-CD3 (Purified NA/LE hamster anti-mouse CD3e, 1 μg/mL, clone 145-2C11) and soluble anti-CD28 (Purified NA/LE hamster anti-mouse CD28, 1 μg/mL, clone 37.51) in the presence of IL-2 (Recombinant mouse IL-2, 20 ng/mL; all reagents from BD Biosciences). Cells were re-stimulated with phorbol-12-myristate-13-acetate (PMA; 50 ng/mL) and ionomycin (1 µg/mL; both from Sigma-Aldrich) for the last 2 (the evaluation of cell proliferation) or 5 h before harvest (the remaining assays). Brefeldin A (Protein transport inhibitor, 1 µL/mL; BD Biosciences) was added to cultures designed to evaluate intracellular cytokine production for the final 4 h to inhibit cytokine release of cytokines and permit their accumulation intracellularly. In turn, cell proliferation was evaluated in the presence of 5-bromo-2′-deoxyuridine (BrdU; APC BrdU Flow Kit, BD Biosciences) at a final concentration of 100 µM in cell culture medium during the last 12 h. Each experiment included 4–5 wells of lymphocytes (obtained from individual mice) for each condition tested. To eliminate the influences of individual differences of animals, the same cells were used as both control and treated cells. All experiments were repeated independently two or three times. The plates were incubated at 37 ºC in an atmosphere of humidified incubator with 5% CO2 and 95% air.

### 4.4. Flow Cytometry

#### 4.4.1. Extracellular Staining

Cells were stained for surface antigens with fluorochrome conjugated monoclonal antibodies (mAb) specific to mouse CD4, CD25, CD49b (all from BD Biosciences, East Rutherford, NJ, USA), and CD39 (BioLegend), as demonstrated in Table 1. After 30 min incubation (on ice and in the dark), the cells were washed in 2 mL of FACS buffer (FB, 1 × Dulbecco’s PBS (Sigma-Aldrich) devoid of Ca^2+^ and Mg^2+^ with 2% (*v*/*v*) heat-inactivated FBS). The staining combinations, properties of antibodies used in the experiments, and evaluated parameters are summarized in Table 1.

#### 4.4.2. Intracellular Staining for Determination of Treg and Tr1 Cells

Cells stained for surface markers (i.e., for CD4 and CD25) as described above were washed, fixed, and permeabilized using a mouse Foxp3 buffer set (BD Biosciences) according to the manufacturer’s protocol. Subsequently, cells were labeled with mAb specific to Foxp3 and CD223 (BD Biosciences). After 45 min incubation (at room temperature), the cells were washed twice with 2 mL of FB and analyzed by flow cytometry.

#### 4.4.3. Intracellular Staining for Determination of IL-10- and TGF-β-Producing Treg and aTeff

Cells stained for surface markers (i.e., for CD4 and CD25) as described above were fixed and permeabilized using Cytofix/Cytoperm solution and Perm/Wash buffer (both from BD Biosciences) according to the manufacturer’s protocol. Subsequently, the samples were stained with mAb specific to Foxp3, IL-10, and TGF-β (BD Biosciences). After 1 h of incubation (at room temperature in the dark) the cells were washed twice with 2 mL of FB and analyzed by flow cytometry.

#### 4.4.4. Intracellular Staining for Determination of Proliferating Treg and aTeff Cells

Cells were stained for Treg markers (i.e., for CD4, CD25, and Foxp3) as described in Section 4.4.2 and thereafter labeled for incorporated BrdU according to the manufacturer’s procedure (APC BrdU Flow Kit, BD Biosciences).

### 4.5. FACS Acquisition and Data Analysis

**Figure 10:**
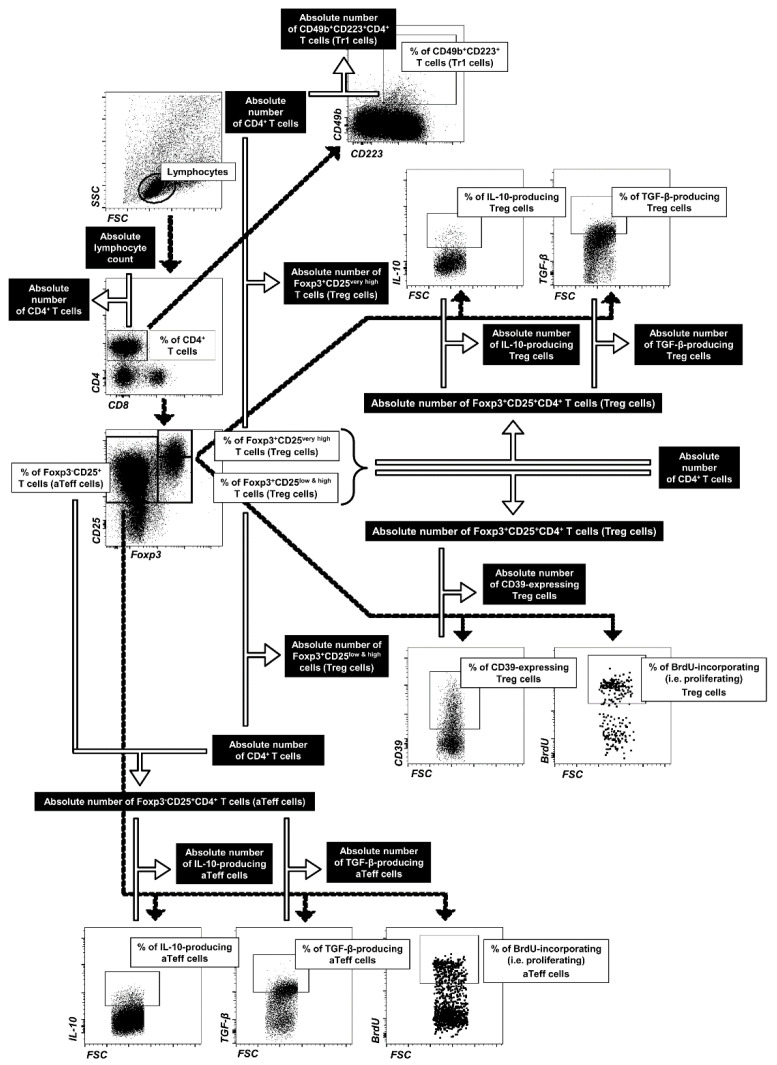
Gating strategy for flow cytometric data analysis and calculation of the absolute cell counts of lymphocyte subsets. Lymphocytes were identified based on forward and side scatter (FSC/SSC) properties, and then gated for expression of CD4 surface receptor. CD4^+^ T cells were analyzed for expression/co-expression of CD25 and Foxp3. On this basis, Foxp3^+^CD25^+^CD4^+^ and Foxp3^−^CD25^+^CD4^+^ T cells, i.e., regulatory (Treg) and activated effector (aTeff) T cells, respectively, were distinguished. Subsequently, IL-10- and TGF-β-producing cells as well as 5-bromo-2-deoxyuridine(BrdU)-incorporating cells were identified within these cell subsets. Moreover, CD39-expressing cells were identified within Treg cell subset. In addition, type 1 regulatory T (Tr1) cells were detected on the basis of CD49b and CD223 co-expression within CD4^+^ T cell subset. Absolute cell counts of lymphocyte subsets (i.e., number of cells from particular subpopulations per sample well) were calculated using the dual platform method, as shown above.

Flow cytometry analysis was performed using a FACSCelesta cytometer (BD Biosciences). The data were acquired by FACSDiva version 9.0 software (BD Biosciences) and analyzed by FlowJo software (Tree Star Inc., Stanford, CA, USA). Absolute cell counts (i.e., number of cells from a particular subpopulation per sample well) were calculated using the dual platform method, i.e., an absolute cell count was determined by calculating the data obtained from a cell counting chamber by the percentage of particular cell subsets, as illustrated in Figure 10.

### 4.6. Statistical Analyses

Results were expressed as the mean (±S.D.) of two or three independent experiments. Statistical analysis was performed using one-way analysis of variance followed by the Bonferroni’s post hoc test. Differences were deemed significant when the *p* values were <0.05. SigmaPlot Software Version 12.0 (Systat Software Inc., San Jose, CA, USA) was used for statistical analysis and the plotting of graphs.

### 4.7. The Approach to Interpreting the Absence/Presence of Interactions between the Evaluated Agents and Types of These Interactions

Traditionally, pharmacodynamic interactions fall into a few types (i.e., superadditivity, additivity, and antagonism) and are assigned simple definitions, whereas in reality they are a more complex phenomenon. This complexity lies primarily in that two substances which—when applied separately—produce an effect in a specific direction or do not produce any effect, may give rise to different types of interaction or may not interact with one another when used together. Hence, the data on effects produced by certain substances administered alone do not let us predict with high certainty what type of interaction if any will occur following their combined application. An exception is the situation where two agents used separately exert opposite effects, as then their combined use will invariably lead to antagonism; however, it should be noted that the extent of such an antagonistic interaction may vary and cannot be predicted based on the effects produced by the agents causing this interaction when administered singly. In order to clarify the complexity of the discussed problem, it was shown in a tabular form (Table 2), including possible interactions between two agents and interpretation of these interactions. Thus, this table is an interpretative key, which helped to identify the absence/presence of interactions between the studied agents and types of these interactions. The definitions of additivity, synergism, and enhancement are in line with the content of the widely cited paper by Toews and Bylund [83] concerning pharmacodynamic interactions. In the light of the results obtained in this study, it was necessary to define an interaction consisting in two agents not affecting a given parameter when applied alone but altering it when used in a combination. To the author’s best knowledge, the literature lacks a definition of this type of interaction, which is why he proposed to call it ‘induction of an effect’ for the purposes of this study. As shown in the table, the basis for drawing a conclusion on the absence/presence of an interaction between two agents, depending on a situation, was the absence/presence of statistically significant differences in the size of an effect: (a) caused by the combination of the two agents versus the effect caused by separate applications of these agents (points 1 and 2 of Table 2); (b) caused by the combination of the two agents versus the control value (points 3 and 4 of Table 2). When an interaction between two agents affecting a given parameter in the same direction was identified, a further step was to classify this interaction into the correct subcategory, i.e., synergism or additivity or subadditivity. To achieve this, it was necessary to determine whether the effect produced by the combination of the two agents was greater, smaller, or equal in comparison with the one that is the sum of effects produced by these agents used separately. To this end, numerical data presenting ‘pure effects’ of the studied agents and their combinations were needed. They were obtained by subtracting the average control value of individual parameters from the appropriate average values obtained for the agents used alone and in the combinations (Supplementary Material Tables S1 and S2). This made it possible to calculate so-called ‘theoretically expected means’, i.e., the effect constituting the sum of the individual effects of the agents alone. These data enabled making the aforementioned comparison, that is, classifying an interaction as synergism or additivity or subadditivity. Although these types of interactions did not appear often, for a more complete picture it was decided to provide in this paper such data as the experimental means obtained after subtracting of control values and ‘theoretically expected means’ achieved for all analyzed parameters (Supplementary Material Tables S1 and S2). Overview tables summarizing the effects of single agents and their combinations on the evaluated parameters and the presence or absence of interactions between these agents were compiled in order to provide a big picture of this research study (Supplementary Material Tables S3 and S4).

## 5. Conclusions

The tested agents exerted the following desired effects: (a) IL-27 induced CD39 expression on Treg cells, the generation of Tr1 cells, and the ability of aTeff cells to IL-10 production; (b) TER induced Foxp3-expressing CD4^+^ T cells and up-regulated density of CD25 on these cells; TER also induced the ability of Treg and aTeff cells to TGF-β production; (c) ATRA induced CD39 expression on Treg cells. The study revealed a strong superadditive effect between IL-27 and ATRA with respect to increasing CD39 expression on Treg cells. IL-27 and ATRA in combination, but not alone, induced the ability of Treg cells to IL-10 production. However, the combination of IL-27, TER, and ATRA did not induce the generation of Treg cell subset with all described above attributes. This was due to the fact that TER abolished all desired effects induced by IL-27 and ATRA. Thus, in the aspect of pharmacological induction of Treg cells with a highly suppressive phenotype the triple combination treatment with TER, IL-27, and ATRA does not provide any benefits over TER alone or dual combination including IL-27 and ATRA. Moreover, it was found that TER fully, while ATRA partially, antagonized IL-27-mediated induction of Tr1 cells.

## 6. Limitation of the Study

The current results must be approached with a good measure of caution until they can be verified in in vivo studies. The in vitro conditions do not reproduce either the pharmacokinetic processes in which a drug is submitted to in a living body, or the regulatory mechanisms which can be activated by the body in response to the tested agents. Therefore, further studies under in vivo conditions are necessary to confirm the findings of the present study and determine their therapeutic potential.

## Figures and Tables

**Table 1:** The staining combinations, characteristics of monoclonal antibodies and evaluated parameters.

Marker	Fluorochrome	Clone	Isotype	Evaluated Parameters
CD4	PerCP-Cy 5.5	clone RM4-5	IgG2a, κ	The percentage and absolute count of: Foxp3^+^CD25^+^CD4^+^, Foxp3^+^CD25^very high^ CD4^+^ and Foxp3^+^CD25^high&low^ T cells (Treg cells and their subsets) CD39^+^Foxp3^+^CD25^+^CD4^+^ T cells (CD39-expressing Treg cells) CD49b^+^CD223^+^CD4^+^ T cells (Tr1 cells)
CD25	PE-Cy7	clone PC61	IgG1, λ	
Foxp3	Alexa Fluor 488	MF23	IgG2b	
CD39	PE	Duha59	IgG2a, κ	
CD49b	PE-CF594	DX5	IgM, κ	
CD223	APC	C9B7W	IgG1, κ	
CD4	PerCP-Cy 5.5	RM4-5	IgG2a, κ	The percentage and absolute count of: IL-10^+^Foxp3^+^CD25^+^CD4^+^ T cells (IL-10-producing Treg cells) IL-10^+^Foxp3^−^CD25^+^CD4^+^ T cells (IL-10-producing aTeff cells) TGF-β^+^Foxp3^+^CD25^+^CD4^+^ T cells (TGF-β-producing Treg cells) TGF-β ^+^Foxp3^−^CD25^+^CD4^+^ T cells (TGF-β-producing aTeff cells)
CD25	PE-Cy7	PC61	IgG1, λ	
Foxp3	Alexa Fluor 488	MF23	IgG2b	
IL-10	APC	JES5-16E3	IgG2b	
TGF-β	PE	TW7-16B4	IgG1, λ	
CD4	FITC	H129.19	IgG2a, κ	The percentage of: BrdU^+^Foxp3^+^CD25^+^CD4^+^ T cells (proliferating Treg cells) BrdU^+^Foxp3^+^CD25^+^CD4^+^ T cells (proliferating Treg cells)
CD25	PE-Cy7	PC61	IgG1, λ	
Foxp3	PE	MF23	IgG2b	
BrdU	APC	IgG2a	BU1/75	

**Table 2:** Overview table of possible types of interactions in the aspect of the present studies.

Description of the Effect of Two Drugs on Evaluated Parameter	Interpretation and Comment
**(1) Agents A and B applied alone have unidirectional action, i.e., change the value of a given parameter in the same direction:**	
Effect of the combination of agent A and agent B is greater than the sum of the individual effects of these agents alone	Superadditivity: synergism (1 + 1 > 2)
Effect of the combination of agent A and agent B constitutes the sum of the individual effects of these agents alone	Additivity (1 + 1 = 2)
Effect of the combination of agent A and agent B is lower than the sum of the individual effects of these agents alone, but greater than the effect of this agent, which exerts a stronger effect	Subadditivity (1 + 1 > 1 but <2)
Effect of the combination of agent A and agent B is the same as that induced by this agent, which exerts a stronger effect	Absence of an interaction (1 + 2 = 2)
Effect of the combination of agent A and agent B is the same as the individual effects of these agents alone (the *overlapping effects of* both agents)	Absence of an interaction (1 + 1 = 1)
**(2) Agent A applied alone acts in a given direction on a given parameter while agent B applied alone does not affect this parameter:**	
Effect of the combination of agent A and agent B is the same as the individual effect of agent A alone	Absence of an interaction (1 + 0 = 1)
Effect of the combination of agent A and agent B is greater than the individual effect of agent A alone (agent B potentiates the effect of agent A, although the former alone does no exert such effect)	Superadditivity: enhancement (1 + 0 > 1)
Effect of the combination of agent A and agent B is lower than the individual effect of agent A alone (agent B antagonizes the effect induced by agent A)	Antagonism (1 + 0 < 1)
**(3) Agents A and B applied alone do not affect the value of a given parameter**:	
Effect of the combination of agent A and agent B is not significantly different from the control value	Absence of an interaction (0 + 0 = 0)
Effect of the combination of agent A and agent B is significantly different from the control value	Induction of an effect (0 + 0 > 0)
**(4) Agents A and B applied alone have opposite actions, i.e., change the value of a given parameter in the opposite direction:**	
Effect of the combination of agent A and agent B does not differ significantly from the control value (the effects of both agents cancel each other out)	Antagonism (1 + −1 = 0)
Effect of the combination of agent A and agent B is lower than the individual effect of agent A alone but is significant, i.e., agent A-induced effect is still significantly different from the control level (the one agent reduces/attenuates, but not fully abolishes, the effect induced by the other one)	Antagonism (1 + −1 > 0)
Effect of the combination of agent A and agent B is the same as the individual effect of one of the agents alone (the *effect of the one agent is fully* manifested, and thus the effect of the other one is abolished)	Antagonism (1 + −1 = −1 or 1 + −1 = 1)

## Data Availability

The data presented in this study are available on reasonable request from the corresponding author.

## Supplementary Materials

The following supporting information can be downloaded at: https://www.mdpi.com/article/10.3390/molecules27238471/s1, Table S1: A summary table showing the treatment means obtained in the experiments and calculated by summing up the means of single agents alone; Table S2: A summary table showing the treatment means obtained in the experiments and calculated by summing up the means of single agents alone; Table S3: Overview table summarizing the effects of single agents and their combinations on the evaluated parameters and the presence or absence of interactions between theses agents; Table S4: Overview table summarizing the effects of particular concentrations of IL-27 and ATRA and their combinations on the evaluated parameters, and the presence or absence of interactions between these agents.
