# Supplementary material for: Effect of IL-27, Teriflunomide and Retinoic Acid and Their Combinations on CD4+ T Regulatory T Cells—An In Vitro Study
Source: Molecules. 2022 Dec 2;27(23):8471. doi: 10.3390/molecules27238471 (PMC9739213; doi:10.3390/molecules27238471)
Supplement: Supplementary file 1 [file molecules-27-08471-s001.zip › molecules-2041019-Supplementary.pdf]

**Effect of IL-27, teriflunomide and retinoic acid and their combinations on CD4+ T regulatory T cells – an in vitro study**

**Tomasz Maślanka**

## Supplementary Materials

**Suppl. Table S1.** A summary table showing the treatment means obtained in the experiments and calculated by summing up the means of single agents alone.

| Parameter | The experimental means obtained after subtracting the control values | | | | | | | The means of treatment combinations calculated by summing up the means of single agents alone ("Theoretically expected means") | | | |
|---|---|---|---|---|---|---|---|---|---|---|---|
| | IL-27 | TER | ATRA | IL-27 + TER | IL-27 + ATRA | TER + ATRA | IL-27 + TER + ATRA | IL-27 + TER | IL-27 + ATRA | TER + ATRA | IL-27 + TER + ATRA |
| **Absolute number of CD4$^+$ T cells - stimulated cultures** | -92292 | -353563 | -173904 | -414107 | -140254 | -395683 | -427925 | -445854 | -266196 | -527467 | -619758 |
| **Absolute number of CD4$^+$ T cells - unstimulated cultures** | 94356 | -176169 | -141438 | -97225 | 10188 | -242269 | -141106 | -81813 | -47081 | -317606 | -223250 |
| **% of Foxp3$^+$CD25$^+$ cells within CD4$^+$ T cells (Treg cells) – stimulated cultures** | 1.21 | 4.27 | -1.21 | 4.03 | -0.16 | 5.24 | 5.02 | 5.47 | 0.00 | 3.06 | 4.27 |
| **Absolute number of Foxp3$^+$CD25$^+$CD4+ T (Treg) cells – stimulated cultures** | 917 | -11012 | -17255 | -17873 | -9688 | -13267 | -17465 | -10095 | -16338 | -28267 | -27350 |
| **% of Foxp3$^+$CD25$^+$ cells within CD4$^+$ T cells (Treg cells) – unstimulated cultures** | -5.74 | 6.73 | -1.20 | -4.18 | -9.05 | 2.21 | -6.48 | 0.99 | -6.94 | 5.53 | -0.21 |
| **Absolute number of Foxp3$^+$CD25$^+$CD4$^+$ (Treg) cells – unstimulated cultures** | -19492 | -3343 | -21543 | -27521 | -40393 | -26223 | -38034 | -22835 | -41035 | -24886 | -44378 |
| **% of CD39$^+$ cells within Treg cells – stimulated cultures** | 3.72 | -5.37 | 4.73 | -3.21 | 21.88 | -4.00 | -0.86 | -1.65 | 8.45 | -0.63 | 3.08 |
| **Absolute number of CD39$^+$ Treg cells – stimulated cultures** | 1647 | -2642 | -555 | -2413 | 5758 | -2323 | -1853 | -994 | 1093 | -3196 | -1549 |
| **% of CD39$^+$ cells within Treg cells – unstimulated cultures** | 0.90 | 0.08 | 0.14 | 0.52 | 0.99 | 0.16 | 0.69 | 0.98 | 1.03 | 0.21 | 1.11 |
| **Absolute number of CD39$^+$ Treg cells – unstimulated cultures** | 277 | 25 | -20 | 77 | 52 | -35 | 24 | 302 | 257 | 5 | 282 |
| **% of IL-10-producing cells within Treg cells** | 0.36 | -1.89 | 0.15 | -0.97 | 2.76 | -1.55 | -0.76 | -1.53 | 0.51 | -1.73 | -1.38 |
| **Absolute number of IL-10-producing Treg cells** | 51 | -865 | -373 | -784 | 667 | -853 | -745 | -814 | -322 | -1239 | -1187 |
| **% of IL-10-producing cells within aTeff cells** | 2.68 | 0.26 | -0.33 | 1.02 | 1.75 | 0.24 | 1.20 | 2.94 | 2.35 | -0.07 | 2.61 |
| **Absolute number of IL-10-producing aTeff cells** | 12582 | -3406 | -1980 | -2842 | 8961 | -4121 | -2579 | 9176 | 10602 | -5387 | 7195 |
| **% of TGF-β-producing cells within Treg cells** | -4.84 | 6.70 | -0.68 | 5.97 | -3.81 | 8.39 | 8.64 | 1.86 | -5.52 | 6.02 | 1.18 |
| **Absolute number of TGF-β-producing Treg cells** | -1955 | 166 | -1610 | -1073 | -1867 | -144 | -348 | -1789 | -3564 | -1444 | -3398 |
| **% of TGF-β-producing cells within aTeff cells** | -1.25 | 15.62 | -1.21 | 12.64 | -1.23 | 14.02 | 11.46 | 14.37 | -2.46 | 14.41 | 13.16 |
| **Absolute number of TGF-β-producing aTeff cells** | -7509 | 18886 | -6722 | 8655 | -5885 | 7965 | 6818 | 11377 | -14231 | 12165 | 4656 |
| **% of BrdU-incorporating Treg cells** | -15.88 | -31.68 | -32.58 | -36.25 | -32.24 | -43.68 | -42.47 | -47.56 | -48.47 | -64.26 | -80.15 |
| **% of BrdU-incorporating aTeff cells** | 3.03 | -16.91 | -10.53 | -17.46 | -7.04 | -17.79 | -18.10 | -13.88 | -7.50 | -27.44 | -24.41 |
| **% of CD49$^+$CD223$^+$ among CD4$^+$ T cells (Tr1 cells)** | 0.53 | -0.19 | -0.03 | -0.12 | 0.17 | -0.21 | -0.18 | 0.35 | 0.51 | -0.21 | 0.32 |
| **Absolute number of CD49$^+$CD223$^+$CD4$^+$ T cells (Tr1 cells)** | 2515 | -1812 | -729 | -1776 | 446 | -1959 | -1919 | 704 | 1787 | -2540 | -25 |

**Suppl. Table S2.** A summary table showing the treatment means obtained in the experiments and calculated by summing up the means of single agents alone.

| **Parameter** | **The experimental means obtained after subtracting the control values** | | | | | | | | **The means of treatment combinations calculated by summing up the means of single agents alone ("Theoretically expected means")** | | | |
|---|---|---|---|---|---|---|---|---|---|---|---|---|
| | IL-27 100 ng | IL-27 10 ng | ATRA $10^{-6}$ | ATRA $10^{-7}$ | IL-27 100 ng + ATRA $10^{-6}$ | IL-27 100 ng + ATRA $10^{-7}$ | IL-27 10 ng + ATRA $10^{-6}$ | IL-27 10 ng + ATRA $10^{-7}$ | IL-27 100 ng + ATRA $10^{-6}$ | IL-27 100 ng + ATRA $10^{-7}$ | IL-27 10 ng + ATRA $10^{-6}$ | IL-27 10 ng + ATRA $10^{-7}$ |
| **% of $CD39^{+}$ cells within Treg cells** | 6.09 | 3.35 | 6.97 | 6.40 | 23.45 | 13.51 | 18.19 | 13.27 | 13.06 | 12.49 | 10.32 | 9.75 |

**Suppl. Table S3.** Overview table summarizing the effects of single agents and their combinations on the evaluated parameters and the presence or absence of interactions between theses agents.

<table>
<tr><th rowspan="3">Parameter</th><th colspan="3" rowspan="2">Effect of single agents on evaluated parameter</th><th colspan="4">Effect of combinations of agents on evaluated parameter/Presence or absence of interactions</th></tr>
<tr><th colspan="3">Dual combinations compared to single agents alone</th><th>Triple combination compared to the dual combinations</th></tr>
<tr><th>IL-27</th><th>TER</th><th>ATRA</th><th>IL-27 + TER</th><th>IL-27 + ATRA</th><th>TER + ATRA</th><th>IL-27 + TER + ATRA</th></tr>
<tr><td>Absolute number of CD4$^+$ T cells - stimulated cultures</td><td>NSS effect</td><td>Decrease</td><td>Decrease</td><td>Decrease/Absence of an interaction</td><td>Decrease/Absence of an interaction</td><td>Decrease/Absence of an interaction</td><td>Decrease/Absence of an interaction</td></tr>
<tr><td>Absolute number of CD4$^+$ T cells - unstimulated cultures</td><td>NSS effect</td><td>Decrease</td><td>Decrease</td><td>NSS effect/Absence of an interaction</td><td>NSS effect/Antagonism of ATRA-induced decrease by IL-27</td><td>Decrease/Absence of an interaction</td><td>Decrease/Absence of an interaction</td></tr>
<tr><td>% of Foxp3$^+$CD25$^+$ cells within CD4$^+$ T cells (Treg cells) - stimulated cultures</td><td>NSS effect</td><td>Increase</td><td>NSS effect</td><td>Increase/Absence of an interaction</td><td>NSS effect/Absence of an interaction</td><td>Increase/Absence of an interaction</td><td>Increase/Absence of an interaction</td></tr>
<tr><td>Absolute number of Foxp3$^+$CD25$^+$CD4+ T (Treg) cells - stimulated cultures</td><td>NSS effect</td><td>Decrease</td><td>Decrease</td><td>Decrease/Absence of an interaction</td><td>Decrease/Absence of an interaction</td><td>Decrease/Absence of an interaction</td><td>Decrease/Absence of an interaction</td></tr>
<tr><td>% of Foxp3$^+$CD25$^+$ cells within CD4$^+$ T cells (Treg cells) - unstimulated cultures</td><td>Decrease</td><td>Increase</td><td>NSS effect</td><td>Decrease/Antagonism of TER-induced increase by IL-27</td><td>Decrease/Superadditivity: enhancement → ATRA enhances IL-27-induced decrease</td><td>Increase/Antagonism of TER-induced increase by ATRA</td><td>Decrease/Antagonism of IL-27 + ATRA-induced enhancement by TER & Superadditivity: enhancement → ATRA enhances the antagonistic effect of IL-27 on TER-induced increase</td></tr>
<tr><td>Absolute number of Foxp3$^+$CD25$^+$CD4$^+$ (Treg) cells - unstimulated cultures</td><td>Decrease</td><td>NSS effect</td><td>Decrease</td><td>Decrease/Absence of an interaction</td><td>Decrease/Additivity</td><td>Decrease/Absence of an interaction</td><td>Decrease/Absence of an interaction</td></tr>
<tr><td>% of CD39$^+$ cells within Treg cells - stimulated cultures</td><td>Increase</td><td>Decrease</td><td>Increase</td><td>Decrease/Antagonism of IL-27-induced increase by TER</td><td>Increase/Superadditivity: synergism</td><td>Decrease/Antagonism of ATRA-induced increase by TER</td><td>NSS effect/Antagonism of IL-27 + ATRA-induced synergism by TER</td></tr>
<tr><td>Absolute number of CD39$^+$Treg cells – stimulated cultures</td><td>Increase</td><td>Decrease</td><td>NSS effect</td><td>Decrease/Antagonism of IL-27-induced increase by TER</td><td>Increase/Superadditivity: enhancement → ATRA enhances the IL-27-induced increase</td><td>Decrease/Absence of an interaction</td><td>Decrease/Antagonism of IL-27 and ATRA-induced enhancement by TER</td></tr>
<tr><td>% of CD39$^+$ cells within Treg cells – unstimulated cultures</td><td>Increase</td><td>NSS effect</td><td>NSS effect</td><td>Increase/Antagonism of IL-27-induced increase by TER</td><td>Increase/Absence of an interaction</td><td>NSS effect/Absence of an interaction</td><td>Increase/Absence of an interaction</td></tr>
<tr><td>Absolute number of CD39$^+$Treg cells – unstimulated cultures</td><td>Increase</td><td>NSS effect</td><td>NSS effect</td><td>NSS effect/Antagonism of IL-27-induced increase by TER</td><td>NSS effect/Antagonism of IL-27-induced increase by ATRA</td><td>NSS effect/Absence of an interaction</td><td>NSS effect/Absence of an interaction</td></tr>
<tr><td>% of IL-10-producing cells within Treg cells</td><td>NSS effect</td><td>Decrease</td><td>NSS effect</td><td>NSS effect/Absence of an interaction</td><td rowspan="2">Increase/Induction of an effect → combination of IL-27 + ATRA induces significant increase in both parameters</td><td rowspan="2">Decrease/Absence of an interaction</td><td>NSS effect/Antagonism of IL-27 + ATRA-induced induction effect by TER & Antagonism of TER-induced decrease by IL-27 + ATRA</td></tr>
<tr><td>Absolute number of IL-10-producing Treg cells</td><td>NSS effect</td><td>Decrease</td><td>NSS effect</td><td>Decrease/Absence of an interaction</td><td>Decrease/Antagonism of IL-27 + ATRA-induced induction effect by TER</td></tr>
</table>

| | | | | | | | |
|---|---|---|---|---|---|---|---|
| % of IL-10-producing cells within aTeff cells | Increase | NSS effect | NSS effect | NSS effect/Antagonism of IL-27-induced increase by TER | Increase/Absence of an interaction | NSS effect/Absence of an interaction | NSS effect/Absence of an interaction |
| Absolute number of IL-10-producing aTeff cells | Increase | NSS effect | NSS effect | | | Decrease/Induction of an effect → combination of TER + ATRA induces significant decrease | NSS effect/ Absence of an interaction |
| % of TGF-β-producing cells within Treg cells | NSS effect | Increase | NSS effect | Increase/Absence of an interaction | NSS effect/Absence of an interaction | Increase/Absence of an interaction | Increase/Absence of an interaction |
| Absolute number of TGF-β-producing Treg cells | Decrease | NSS effect | Decrease | NSS effect/Antagonism of IL-27-induced decrease by TER | Decrease/Absence of an interaction (the same effects of both agents overlap) | NSS effect/Antagonism of ATRA-induced decrease by TER | NSS effect/ Antagonism of IL-27 + ATRA-induced decrease by TER |
| % of TGF-β-producing cells within aTeff cells | NSS effect | Increase | NSS effect | Increase/Absence of an interaction | NSS effect/Absence of an interaction | Increase/Absence of an interaction | Increase/Induction of an effect → combination of IL-27 and ATRA antagonizes, but to a very limited extent, the TER-induced increase |
| Absolute number of TGF-β-producing aTeff cells | NSS effect | Increase | NSS effect | Increase/Antagonism of TER-induced increase by IL-27 | NSS effect/Absence of an interaction | NSS effect/Antagonism of TER-induced increase by ATRA | NSS effect/ Absence of an interaction |
| % of BrdU-incorporating Treg cells | NSS effect | Decrease | Decrease | Decrease/Absence of an interaction | Decrease/Absence of an interaction | Decrease/Absence of an interaction | Decrease/Absence of an interaction |
| % of BrdU-incorporating aTeff cells | NSS effect | Decrease | Decrease | Decrease/Absence of an interaction | NSS effect/Absence of an interaction | Decrease/Absence of an interaction | Decrease/Absence of an interaction |
| % of $CD49^{+}CD223^{+}$ within $CD4^{+}$ T cells (Tr1 cells) | Increase | Decrease | NSS effect | NSS effect/Antagonism of IL-27-induced increase by TER & Antagonism of TER-induced decrease by IL-27 | Increase/Antagonism of IL-27-induced increase by ATRA | Decrease/Absence of an interaction | Decrease/Absence of an interaction |
| Absolute number of $CD49^{+}CD223^{+}CD4^{+}$ T cells (Tr1 cells) | Increase | Decrease | NSS effect | Decrease/Antagonism of IL-27-induced increase by TER | NSS effect/Antagonism of IL-27-induced increase by ATRA | | |

NSS: not statistically significant

**Suppl. Table S4.** Overview table summarizing the effects of particular concentrations of IL-27 and ATRA and their combinations on the evaluated parameters, and the presence or absence of interactions between these agents.

| **Parameter** | **Effect of single agents on evaluated parameter** | | | | **Dual combinations compared to single agents alone** | | | |
|---|---|---|---|---|---|---|---|---|
| | **IL-27 100 ng** | **IL-27 10 ng** | **ATRA $10^{-6}$** | **ATRA $10^{-7}$** | **IL-27 100 ng + ATRA $10^{-6}$** | **IL-27 100 ng + ATRA $10^{-7}$** | **IL-27 10 ng + ATRA $10^{-6}$** | **IL-27 10 ng + ATRA $10^{-7}$** |
| **% of $CD39^{+}$ cells within Treg cells** | Increase | NSS effect | Increase | Increase | Superadditivity: synergizm | Small scale superadditivity: synergism | Superadditivity: enhancement | Superadditivity: enhancement |

NSS: not statistically significant
